# Effects of Perceived Scarcity on Mental Health, Time and Risk Preferences, and Decision-Making During and After COVID-19 Lockdown: Quasi-Natural Experimental Study

**DOI:** 10.2196/69496

**Published:** 2025-08-29

**Authors:** Haiou Zhu, Fangzhou You, Thorsten Gruber, Hua Dong, Cees de Bont

**Affiliations:** 1 Department of Psychiatry, University of Oxford, Warneford Hospital, Warneford Lane, Headington, Oxford, OX3 7JX, United Kingdom, 44 07754113721; 2 School of Design and Creative Arts, Loughborough University, Loughborough, United Kingdom; 3 School of Arts and Creative Technologies, University of York, York, United Kingdom; 4 Centre for Service Management, Business School, Loughborough University, Loughborough, United Kingdom; 5 Brunel Design School, Brunel University London, London, United Kingdom; 6 College of Design and Engineering, National University of Singapore, Singapore, Singapore

**Keywords:** perceived scarcity, COVID-19 lockdown, mental health, wellbeing, cognitive function, present bias, risk aversion, natural experiment, public health interventions, consumer behavior

## Abstract

**Background:**

The COVID-19 lockdowns led to significant resource constraints, potentially impacting mental health and decision-making behaviors. Understanding the psychological and behavioral consequences could inform designing interventions to mitigate the negative impacts of episodic scarcity during crises like pandemics.

**Objective:**

To investigate the effects of perceived scarcity on mental health (stress and fear), cognitive functioning, time and risk preferences (present bias and risk aversion), and trade-offs between groceries, health, and temptation goods during and after the COVID-19 lockdown in Shanghai.

**Methods:**

A quasi-natural experiment was conducted in Shanghai during and after the COVID-19 lockdown. Web-based surveys were administered in May 2022 (during lockdown) and September 2022 (post-lockdown). Propensity score matching was used to balance demographic factors between the groups (During: n=332; After: n=339). Data were analyzed using regression analyses, controlling for potential confounders and applying propensity score matching weights.

**Results:**

Perceived scarcity was significantly higher during the lockdown (mean 7.97 (SD 2.1)) than after (mean 4.35 (SD 2.27); *P*<.001). Higher perceived scarcity was associated with increased stress levels both during (standardized β coefficient=.62, *P*<.001) and after the lockdown (standardized β coefficient=.65, *P*<.001). Perceived scarcity also predicted greater fear of COVID-19 after lockdown (standardized β coefficient =.38, *P*<.001), though not during lockdown. Cognitive functioning remained stable, possibly due to a ceiling effect from high education levels. Monetary risk aversion increased under prolonged scarcity during lockdown (scarcity×during-lockdown interaction standardized β coefficient=4.68, *P*<.001). Present bias (tendency to choose immediate rewards) showed no significant overall change between groups, in line with recent evidence of stable time preferences during the pandemic. During lockdown, participants allocated more budget to groceries (standardized β coefficient=.67, *P*=.01) and less to health items (standardized β coefficient=−.61, *P*=.02), compared to post-lockdown, reflecting shifted priorities on pressing needs under scarcity. Subgroup analyses indicated stratified heterogeneity. Women increased their grocery spending (standardized β coefficient =.16, *P*=.04) and reduced spending on health items (standardized β coefficient = –.15, *P*=.05). Lower education participants exhibited more risk-averse attitudes (standardized β coefficient =.80, *P*=.01) under scarcity, whereas age and income did not significantly moderate these effects.

**Conclusions:**

The study highlights that perceived scarcity during lockdown intensified stress and altered decision-making behaviors, including increased monetary risk aversion and shifts in spending priorities. Theoretically, this study advances the understanding of perceived scarcity by exploring its domain-specific effects on mental health and decision-making. Practically, these findings emphasize the need for public health strategies that mitigate the psychological impact of scarcity during crises, ensure access to essential goods, and support adaptive decision-making behaviors.

## Introduction

Lockdown policies have been among the most effective public health interventions during the COVID-19 pandemic, saving millions of lives worldwide [1,2]. However, lockdown measures, including social distancing, self-isolation, and prolonged home quarantine, along with disruptions to supply chains and shortages of essential products and services, have significantly altered many aspects of people’s daily lives [3,4]. Research has shown that such measures have exacerbated mental health problems, leading to increased stress, depression, and negative affect [5-8], affected job, financial, and food security [9,10], and caused panic buying [11,12] and adverse health decisions and behaviors [13-15]. While many studies have used longitudinal and natural experiments to investigate the impact of lockdowns [16-18], few have focused on the psychological and behavioral consequences arising from the radical resource constraints individuals face during these periods.

A location where a stringent lockdown was implemented in response to a widespread COVID-19 outbreak was Shanghai, China’s largest city by population (over 24 million) and one of the largest cities in the world. The lockdown lasted from March to May 2022, and residents were largely quarantined at home, facing significant shortages of products and services. After the city reopened on June 1, 2022, the supply and delivery of products and services resumed pre-lockdown levels. This abrupt change in resource availability created a unique opportunity to examine how resource scarcity during lockdown affected various aspects of residents’ lives in one of the largest urban populations in the world. The timing of this study is important since it captured a real-world episode of intense scarcity [19,20]. Understanding how these episodic constraints affect mental health and health-related decisions and behaviors can provide valuable empirical evidence on the impacts of pandemic lockdowns.

The concept of perceived scarcity, or psychological scarcity, is rooted in poverty literature and is well-established in the psychology and economics literature [21-23]. It refers to the subjective feelings that arise from the gap between one’s needs and the resources available to fulfill them [24,25]. Feelings of scarcity can preoccupy one’s thoughts, making everyday decisions, such as whether to visit a doctor or buy groceries, more difficult and stressful [26]. While traditionally studied in the context of poverty, recent research suggests that perceived scarcity can affect individuals across socioeconomic statuses, particularly during crises that disrupt normal resource access [27]. Previous studies have linked perceived scarcity to increased stress and negative affect [21,28,29]. Research also suggests that scarcity can directly impair cognitive functions, including decision-making, reasoning, problem-solving, and attention, by reducing cognitive bandwidth and limiting the mental resources necessary to process information and make decisions [24,30]. However, these effects have not been consistently confirmed in other empirical studies [27-29]. Both the negative emotional states and the cognitive load induced by scarcity are seen as mechanisms that cause scarcity-reinforcing decisions and behaviors, leading to a cycle where poor decisions exacerbate conditions of health and well-being [17]. During the COVID-19 pandemic, stress and fear became widespread as individuals faced uncertainty and isolation, along with increasing cognitive load [31-34]. However, it remains unclear how these feelings and cognitive functioning may have been exacerbated by perceived scarcity [35].

Perceived scarcity can also impact decision-making in various domains by inducing biases and trade-off thinking. Time and risk preferences are fundamental components of human decision-making, with present bias and risk aversion representing systematic deviations from rational behavior [36,37]. Present bias refers to the tendency to prioritize immediate rewards over future gains, leading to short-sighted decisions that come at the expense of long-term goals [38,39]. Risk aversion, on the other hand, describes the preference for avoiding risks, making individuals less inclined to engage in actions with uncertain outcomes [40]. Both present bias and risk aversion influence not only financial decisions but also health-related behaviors, such as smoking [41,42] and obesity [43], which are considered irrational. Research on perceived scarcity shows mixed outcomes, with some studies indicating increased risk-taking [44] and time-discounting [45,46], while others find no significant effects [46]. In terms of trade-off thinking, feelings of scarcity induced by financial worries seem to shift attention toward pressing needs, such as food and groceries, at the expense of forward-looking decisions like saving for health and education [47,48]. Additionally, scarcity may lead to the overconsumption of temptation goods, including junk food, sugary drinks, and cigarettes [49]. Nonetheless, these findings are inconsistent, suggesting that the effects of perceived scarcity may vary based on context and population characteristics [22,50]. It remains unclear whether scarcity caused by supply shortages would force individuals to make trade-offs between pressing needs and their health in a pandemic context [3,11,16].

Using a quasi-experimental design, this study aims to examine the effects of perceived scarcity during a COVID-19 lockdown on individuals’ mental health and decision-making, compared with those surveyed after lockdown when scarcity had abated. Specifically, the study investigates whether experiencing resource scarcity is associated with higher stress and fear (mental health outcomes), changes in cognitive function, present bias, or risk aversion (time and risk preferences), and altered spending trade-offs between necessities and non-essentials. The hypothesis was that the lockdown-induced scarcity would heighten stress and fear levels and lead to more cautious decision-making: namely, increased risk aversion, a shift toward present-focused choices, and a reallocation of spending toward essential goods. Furthermore, to estimate the potential cumulative effects of lockdown, the study examines whether the length of exposure to lockdown, ie, the duration of home quarantine and the length of time without shopping, exacerbated mental well-being and influenced decision-making. This study contributes to the literature by extending the concept of perceived scarcity in a high-income, urban population during a crisis, advancing the understanding of its impact on their mental health and decision-making behaviors. The findings can inform policy makers and public health practitioners in designing interventions to mitigate the negative impacts of scarcity during crises and emergencies, ensuring that strategies are tailored to address both supply shortages and psychological well-being.

## Methods

### Study Design

This study adopted the target trial framework [19] for the design, analysis, and reporting of the natural experimental study. The COVID-19 lockdown policy in Shanghai served as the intervention, with the treatment group comprising participants affected during the lockdown, and the control group comprising participants who had resumed regular daily activities by September 2022, 3 months after the city’s reopening. Shanghai was chosen due to its unique and stringent lockdown measures during the COVID-19 pandemic, which lasted from March to May 2022. Shanghai’s lockdown provided an opportunity to study the psychological and behavioral impacts of resource scarcity in an urban setting. According to prior conceptualizations of natural experiment [19,20] and the Medical Research Council guidelines [51,52], this study qualifies as a between-group natural experiment, as it uses the natural occurrence of the lockdown to assign exposure to participants in a manner akin to randomization, which is pivotal for establishing causal relationships in situations where controlled random assignment is not feasible or ethical [53]. While we cannot prove causality definitively, the natural experiment design, treating the lockdown as an exogenous shock, could provide suggestive causal evidence on the impact of perceived scarcity .

### Sample and Data Collection

In total, 2 rounds of web-based surveys with identical items were conducted via Wenjuanxing, a widely used online survey platform in China [54], to collect data from participants living in Shanghai during and after the COVID-19 lockdown. Aligned with the actual lockdown period, which began in late March and ended in late May 2022, the first survey round was distributed in May 2022, near the end of the lockdown, to capture participants’ experiences during quarantine. The second survey round was conducted in September 2022, approximately 3 months after the city had fully reopened, allowing assessment of post-lockdown outcomes. Data collection for each wave was completed within approximately 2‐3 weeks, balancing the urgency to capture the lockdown experience and practical considerations (eg, time and monetary resources available). Figure S1 in Multimedia Appendix 1 illustrates the study setting on a map, highlighting the lockdown context and timeline.

Since the study required recruitment of participants residing specifically in Shanghai during designated timeframes, the survey was distributed by subscribing to targeted sampling services provided by the survey platform (fee details are described below under Ethical Considerations). Recruitment was carefully targeted to ensure participants met the location and timing criteria. Only individuals residing in Shanghai during the designated timeframes were eligible. Participants in the During-lockdown group were asked to confirm that they were currently under home quarantine at the time of the survey and provide the start date of their quarantine, while participants in the After-lockdown group were asked to confirm that they were no longer in quarantine and provide the end date of their quarantine period. Additional quality control measures were used: attention checks embedded within the survey, minimum completion times enforced for each survey section, and systematic screening conducted both automatically by the platform and manually by researchers.

A statistical power analysis, adjusted for multiple comparisons (α≈.0045) to account for all 11 primary outcome measures and targeting a power of 0.80, determined that at least 288 participants per group were required. A total of 747 participants completed the survey, 367 during the lockdown and 380 after the lockdown. The criteria of data exclusion and the number excluded include the following: (1) not in quarantine for During group or still in quarantine for After group (n=23); (2) not living in Shanghai (n=27); and (3) outliers (n=20) who may have been distracted or encountered technical difficulties, and took excessively long to complete the survey (Z-score >mean+3σ, representing the top 0.15% of completion times). After applying these criteria, 70 participants were excluded (n=36 from the “During” group; n=34 from the “After” group), resulting in a sample of 677 participants.

### Propensity Score Matching

To address potential confounding variables and ensure comparability between the “During” and “After” groups, propensity score matching was implemented. Each participant in the “After” group was matched to a participant in the “During” group with a similar propensity score based on demographic variables, including gender, age, education, monthly income, and monthly expenditure. The study used a kernel matching technique (Gaussian kernel, bandwidth =0.06) using the *psmatch2* routine in Stata, which allowed each treated case (During group) to be matched with a weighted composite of control cases (After group) within the kernel bandwidth, maximizing data usage and reducing variance compared to one-to-one nearest-neighbor matching. Matching quality was evaluated using standardized mean differences for each covariate and Rubin B and R statistics. Post-matching balance was satisfactory: all covariate standardized mean differences fell below 0.1 (10%), Rubin B was 8.7% (well under the 25% threshold), and Rubin R was 0.94 (within the recommended 0.5‐2.0 range), indicating a successful match. Visual inspection of the propensity score distribution confirmed substantial overlap between treatment and control groups (see Table S1 and Figure S1 in Multimedia Appendix 2). After matching, 6 participants fell outside the common support and were excluded, leaving 671 participants on support (332 During; 339 After) used in the main analyses.

### Measurements

The measurements for both During and After groups include perceived scarcity, stress, fear of COVID-19, cognitive functioning, time and risk preferences, purchase decisions, and demographic information. Multimedia Appendix 1 gives an overview of the constructs and their measurement items.

For the measurement of perceived scarcity, the study used a 4-item perceived scarcity scale, adapted from Yuen et al [11] and Byun and Sternquist [55]. Items (rated 1=“strongly disagree” to 10=“strongly agree”) measured the extent to which participants felt essential products were in short supply or hard to obtain, with higher scores indicating greater perceived scarcity. Example items include “It was hard to buy enough food and daily supplies” and “Products I needed were often sold out.” Because perceived scarcity may accumulate with prolonged isolation, this study also recorded 2 “exposure” metrics: the start date of home quarantine and the last date the participant went shopping. For the After group, participants provided the date their quarantine ended and the date they went shopping. These metrics allowed examining interaction effects between scarcity perceptions and the length of quarantine or time without shopping.

Participants’ stress levels over the past month were measured using the 10-item Perceived Stress Scale (PSS) from Cohen et al [56]. To measure their fear of COVID-19, the study used the 7-item Fear of COVID-19 Scale from Ahorsu et al [57]. Cognitive performance was evaluated using subset E (consisting of 12 matrix reasoning items), the most difficult of the 5 progressively harder subsets (A-E) in the 60-item Standard Progressive Matrices Test [58]. Participants received 1 point for each correct answer (maximum score=12), and cognitive accuracy was calculated as the proportion of correct responses out of 12. Raven’s matrices are commonly used to assess logical reasoning ability and problem-solving skills [59]. This subset approach was chosen to reduce survey length while still capturing variation in cognitive functioning.

The study measured both monetary-framed and health-framed time preferences using 2 sets of choice scenarios based on Laibson’s quasi-hyperbolic discounting model [39]. In both monetary (job payment) and health (physical examination subsidy) contexts, participants chose between smaller immediate rewards and larger delayed rewards, indicating their level of impatience or present bias [42]. Additional health-related tasks involved choosing between immediate medical attention versus continuing to work for compensation and immediate medical attention versus traveling to a distant hospital. Present bias was identified by the point at which participants switched from preferring later payoffs to earlier ones.

Risk aversion was measured through a series of lottery-choice tasks adapted from the well-established Eckel & Grossman method [60]. In a monetary risk task, participants chose 1 of 6 lotteries, each with a 50/50 chance of a lower or higher payoff, with expected payoffs increasing from the safest (50% chance of winning ¥280 (US$42), 50% chance of winning ¥280 (US$42)) to the riskiest lottery (50% chance of winning ¥700 (US$105), 50% chance of winning ¥20 (US $3)). In a health risk task, participants chose between hypothetical vaccine options with different durations of protection and associated probabilities, adapted from the classic disease problem framework developed by Tversky and Kahneman [61]. Risk aversion was quantified based on the chosen lottery: selecting safer lotteries indicates higher risk aversion. This study chose the Eckel & Grossman approach due to its simplicity for respondents in an online setting and easiness to adapt to both monetary and health scenarios. Note that the study used the non-incentivized elicitation methods for time and risk preferences. The reason was that participants during the lockdown found themselves within a “naturally occurring” state of both financial and health distress. This context improved the likelihood of respondents perceiving the intertemporal and risk trade-offs as more realistic and vivid even in the absence of actual incentive-compatible consequences for their responses.

For the measurement of trade-offs, participants were asked to allocate a hypothetical budget of ¥300 (US $45) over three categories: (1) daily groceries (food and household essentials), (2) health-related goods (medicine and health services), and (3) temptation goods (eg, snacks, toys, or other non-essentials). Each category represents different facets of decision-making, informed by existing literature on trade-off thinking in decision-making [47,49,62]. The budget allocation task was designed to mimic the experience of online shopping, aiming to make the decision-making process feel as realistic as possible for participants, thereby potentially eliciting choices that are representative of their real-world trade-offs and priorities. The items selected in each category were popular and well-known in the local market. The proportion of the budget allocated to each category provides a measure of the importance or priority given to that category, which allows researchers to measure individuals’ decision priority under conditions of scarcity. At the end of the survey, the study collected demographic information, including gender, age, education, monthly income, and monthly expenditure.

### Ethical Considerations

This study was approved by the Ethics Review Committee of Loughborough University (Approval ID: 2022-8493-10095). All participants provided informed consent electronically prior to participation. The consent form informed participants about the study’s purpose, the types of data being collected, and their rights (voluntary participation and ability to withdraw at any time). To protect privacy, no personally identifying information was collected; responses were kept anonymous and only analyzed in aggregate. All data were stored securely with access limited to the research team. No identifiable images or personal data are presented in this paper. Using the platform’s services cost US$1120, equivalent to US$1.5 per participant. Participants did not receive direct monetary compensation; however, Wenjuanxing’s panel service provided a small incentive (equivalent to approximately US$0.9 per participant) as part of its platform reward system.

### Data Analysis

Data were analyzed using Stata 17. The main analysis used ordinary least squares (OLS) regression for continuous outcomes (perceived stress score, fear score, cognitive score, and budget allocation percentages), logistic regression for binary outcomes (eg, whether an individual exhibited present bias), and ordinal logistic regression for ordinal outcomes (risk aversion as lottery choices between 1 and 6). All regressions applied propensity score-matching weights to adjust for baseline differences between the groups. Demographic variables (gender, age, education, income, and expenditure) were included as covariates to control for potential confounding factors. Robust standard errors were used to account for heteroskedasticity and ensure reliable inference. The study probed interaction effects to examine the cumulative impact of lockdown exposure. In particular, the regression models included interaction terms between perceived scarcity and the lockdown group indicator (During vs After) as well as between perceived scarcity and the 2 exposure duration measures (days in quarantine and days since last shopping). These interactions tested whether the relationship between scarcity and outcomes differed by context (lockdown vs post-lockdown) or by how long someone had been in quarantine or without shopping. All continuous variables were mean-centered (or standardized) where appropriate to facilitate interpretation of interactions.

In all OLS regressions, multicollinearity was assessed using variance inflation factors (VIF). Some variables and interaction terms (eg, education and income when both are included, or interactions involving highly correlated measures) showed elevated VIFs (>10). However, given their theoretical importance, we retained them. The mean VIFs for the main regression models ranged around 4.8‐6.3, indicating acceptable multicollinearity levels (see Table S2 in Multimedia Appendix 2 for full diagnostics). Sensitivity analyses were conducted to ensure robustness of results. First, 2-sample tests and non-parametric tests to compare group differences on key outcomes. Results were consistent with the regression analyses (see Table S3 in Multimedia Appendix 2). Second, Tobit models were applied for outcomes with bounded scales (budget allocation proportions), and the findings remained substantively unchanged (see Table S4 in Multimedia Appendix 2). Finally, we explored subgroup analyses by age, gender, education, and income to investigate heterogeneity (see Table S6 in Multimedia Appendix 2).

## Results

### Demographic Characteristics

Table 1 presents the demographic characteristics of participants included in the analysis. The total sample size comprises 671 participants, with 332 in the During group and 339 in the After group. Females comprise 56.6% of the During group and 53.1% of the After group. Males make up 43.4% and 46.9% of these groups, respectively. Most participants are aged 18‐40, with 86.4% in the During group and 89.1% in the After group falling within this range. The majority of participants have an undergraduate degree, 61.7% in the During group and 77.6% in the After group. Graduate degrees or higher are more common in the During group (31.3%) than in the After group (14.5%). The largest income bracket is ¥10,001 (US$1500) and above, representing 54.5% of the During group and 56.9% of the After group. Most participants spend between ¥3001 (US$450) and ¥7000 (US$1050) monthly, with 51.2% in the During group and 56.7% in the After group. These figures indicate that the 2 groups were well-balanced on key demographics.

**Table 1.** Demographic characteristics of study participants by survey group (during lockdown in May 2022 versus after lockdown in September 2022).

Characteristic	During	After	Total
	(n=332)	(n=339)	(N=671)
Gender, n (%)			
Female	188 (56.6)	180 (53.1)	368 (54.8)
Male	144 (43.4)	159 (46.9)	303 (45.2)
Age, n (%)			
18‐30 years	176 (53.0)	199 (58.7)	375 (55.9)
31‐40 years	111 (33.4)	103 (30.4)	214 (31.9)
41‐50 years	42 (12.7)	32 (9.4)	74 (11.0)
51 years and older	3 (0.9)	5 (1.5)	8 (1.2)
Education, n (%)			
Middle school and below	7 (2.1)	10 (2.9)	17 (2.5)
High school	16 (4.8)	17 (5.0)	33 (4.9)
Undergraduate	205 (61.7)	263 (77.6)	468 (69.7)
Graduate and above	104 (31.3)	49 (14.5)	153 (22.8)
Monthly income^a^, n (%)			
¥2000 and below	9 (2.7)	12 (3.5)	21 (3.1)
¥2001-¥4000	13 (3.9)	9 (2.7)	22 (3.3)
¥4001-¥6000	26 (7.8)	15 (4.4)	41 (6.1)
¥6001-¥8000	39 (11.7)	34 (10.0)	73 (10.9)
¥8001-¥10,000	64 (19.3)	76 (22.4)	140 (20.9)
¥10,001 and above	181 (54.5)	193 (56.9)	374 (55.7)
Monthly expenditure, n (%)			
¥1000 and below	7 (2.1)	6 (1.8)	13 (1.9)
¥1001-¥3000	66 (19.9)	63 (18.6)	129 (19.2)
¥3001-¥5000	107 (32.2)	108 (31.9)	215 (32.0)
¥5001-¥7000	63 (19.0)	84 (24.8)	147 (21.9)
¥7001-¥9000	34 (10.2)	48 (14.2)	82 (12.2)
¥9001 and above	55 (16.6)	30 (8.8)	85 (12.7)

a 1 yuan = US$0.15.

### Lockdown Treatment Effects

**Figure 1.**
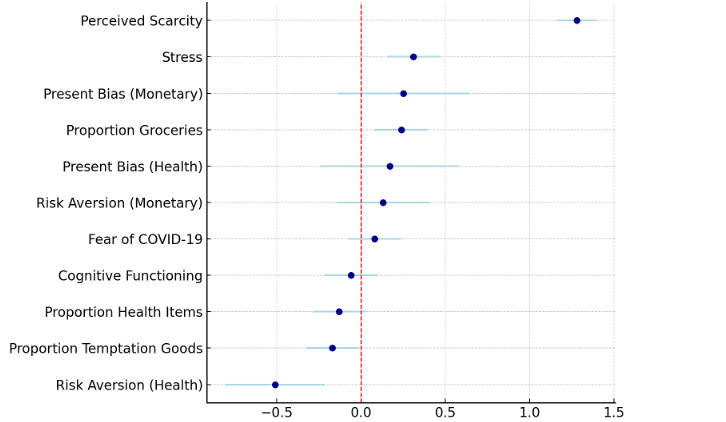
Standardized regression coefficients showing the effects of COVID-19 lockdown (during vs after) on perceived scarcity, stress, fear of COVID-19, time preference (present bias), and risk preference (risk aversion) in monetary- and health-framed choices, and spending trade-offs between grocery, health, and temptation goods. Estimates are plotted with 95% confidence intervals. Each estimate was obtained from ordinary least squares regression except for present bias (logistic regression) and risk aversion (ordinal regression). All regression models applied propensity score-matching weights, controlled for demographic variables, including gender, age, education, monthly income, and monthly expenditure, and used robust standard errors.

Figure 1 shows the estimated effects of lockdown exposure on key outcomes using regression models, which included demographic controls and propensity score matching weights (see Table S5 in Multimedia Appendix 2 for full regression model results). Participants during lockdown reported substantially higher perceived scarcity (mean 7.97 (SD 1.54)) than those after lockdown (mean 4.35 (SD 2.21)) (detailed group comparisons and mean differences for all outcome measures are provided in Table S4 in Multimedia Appendix 2). This difference is significant in OLS regression models (standardized β coefficient =1.28, *P*<.001), indicating that the lockdown created a pronounced sense of resource scarcity. Stress levels were markedly elevated during the lockdown (mean 2.76 (SD 0.70)) versus post-lockdown (mean 2.55 (SD 0.68)) and the increase was significant (standardized β coefficient=.31, *P*<.001). Fear of COVID-19 was slightly higher after the lockdown (mean 4.39 (SD 2.11)) compared to during the lockdown (mean 4.05 (SD 2.06)), though this difference was not significant in regression models. No significant differences were observed in cognitive functioning between the 2 groups in terms of their accuracy on the Raven’s Matrices task (standardized β coefficient= –.06, *P*=.49).

In monetary-framed choices, the proportion of participants exhibiting present bias was 54.3% (119/219) in the During group and 49.1% (111/226) in the After group, with an insignificant difference. Similarly, in health-framed choices, the proportion was 47.0% (103/219) in the During group and 42.5% (96/226) in the After group, and the difference was also not significant. While monetary risk aversion showed a non-significant difference, health-related risk aversion was significantly greater during the lockdown than after (standardized β coefficient =−.51, *P*<.001). Participants during the lockdown allocated a higher proportion of their hypothetical budget to groceries (227/332, 68.3%) compared to those after the lockdown (218/339, 64.2%). This difference was significant in regression results (standardized β coefficient =.24, *P*<.001). Conversely, spending on temptation goods was lower during the lockdown (46/332, 13.9%) than after (56/339, 16.5%), with regression analyses indicating a significant reduction (standardized β coefficient =-.17, *P*=.03). There was no significant difference in the proportion allocated to health-related goods between the 2 groups (standardized β coefficient =.13; *P*=.10).

### Effects of Perceived Scarcity and Lockdown Duration

The cumulative effects of the lockdown were examined by analyzing how perceived scarcity and its interactions with lockdown status and lockdown duration affected various outcomes. To align with the timing of the lockdown, the analysis included participants who had quarantined within 120 days and those who had not shopped in the last 90 days. All variables were normalized to z-scores to facilitate comparison. Table 2 presents the results of regression analyses for the During and After groups. Perceived scarcity was significantly associated with increased stress in both During (standardized β coefficient =.62, *P*<.001) and After (standardized β coefficient =.65, *P*<.001) groups. Neither length of quarantine (days in quarantine for the During group and days after quarantine for the After group) nor days since last shop had significant direct effects on stress. Their interactions with perceived scarcity were also non-significant for both groups. These findings indicate that perceived scarcity remained a robust predictor of stress, independent of quarantine duration. Among participants surveyed during lockdown, perceived scarcity did not significantly affect fear of COVID-19 (standardized β coefficient =.04, *P*=.88), but its interaction with days in quarantine was significantly negative (standardized β coefficient =−1.25, *P*<.001), suggesting that the relationship between scarcity and fear diminished as quarantine duration increased. In the After-lockdown group, perceived scarcity significantly increased fear of COVID-19 (standardized β coefficient=.38, *P*<.001). During lockdown, perceived scarcity was associated with a slight enhancement in cognitive performance (standardized β coefficient =.80, *P*=.04), but as lockdown days extended, cognitive functioning decreased marginally (standardized β coefficient =−4.13, *P*=.06). After lockdown, perceived scarcity had no significant effect on cognitive functioning, while longer days post-lockdown were associated with improved cognitive performance (standardized β coefficient =2.22, *P*=.03).

For both groups, present bias in a monetary context showed limited significant relationships with perceived scarcity and lockdown duration, indicating that scarcity may not substantially alter time preferences in decision-making within these domains (Table 3). Present bias in the health context showed mixed results. For the During group, no significant effect was found. For the After group, days after quarantine significantly reduced health-related present bias (standardized β coefficient =−9.38, *P*=.01), and their interaction with scarcity is also significant (standardized β coefficient =−6.24, *P*=.01). Interaction between perceived scarcity and days since last shopping also significantly reduced health-related present bias (standardized β coefficient =−3.02, *P*=.03). In general, neither perceived scarcity nor lockdown duration significantly influences risk aversion across monetary and health contexts. However, the interaction with days in quarantine is significantly positive (standardized β coefficient =4.68, *P*<.001), indicating scarcity’s impact on increasing monetary risk aversion with longer quarantine durations.

Table 4 presents the regression results for budget allocations to groceries, health items, and temptation goods, assessing trade-off thinking in decision-making. During lockdown, perceived scarcity significantly increased the proportion of the budget allocated to groceries (standardized β coefficient =.67, *P*=.01) and reduced the proportion to health items (standardized β coefficient =−.61, *P*=.02). Even though longer quarantine duration led to decreased grocery spending (standardized β coefficient =−4.43, *P*<.001) and increased health spending (standardized β coefficient =3.98, *P*=.01), its interaction with scarcity significantly increased groceries spending (standardized β coefficient=1.52, *P*=.01) and decreased health spending (standardized β coefficient=−1.41, *P*<.001). In contrast, after lockdown, perceived scarcity and days after lockdown seem to reduce spending on groceries (standardized β coefficient = −0.35, *P*<.001) and increase spending on temptation goods (standardized β coefficient =.29, *P*=.01), while it did not significantly affect expenditures on health items (standardized β coefficient =.29, *P*=.12). But it is important to note that the overall effect sizes were small.

**Table 2.** Effects of perceived scarcity and lockdown duration on stress, fear, and cognitive functioning during and after the COVID-19 lockdown.^a^

Effect	Stress level			Fear of COVID-19			Cognitive functioning		
	b	SE	*P* value	b	SE	*P* value	b	SE	*P* value
Panel A: During lockdown									
Perceived scarcity	0.62	0.22	<.001	0.04	0.28	.88	0.80	0.39	.04
Days since quarantine^b^	−0.89	1.18	.45	1.63	1.53	.29	−4.13	2.15	.06
Perceived scarcity # days since quarantine	−0.47	0.55	.39	−1.25	0.37	<.001	0.64	0.37	.08
Days without shopping^c^	−0.08	0.69	.91	0.94	0.49	.06	1.05	0.57	.07
Perceived scarcity # days without shopping	−0.71	0.51	.17	−0.24	0.31	.44	−0.40	0.33	.23
Panel B: After lockdown									
Perceived scarcity	0.65	0.10	<.001	0.38	0.12	<.001	−0.03	0.12	.78
Days after quarantine	1.27	0.82	.12	−1.34	1.01	.19	2.22	1.00	.03
Perceived scarcity # days after quarantine	0.48	0.53	.37	0.21	0.57	.72	0.97	0.56	.09
Days without shopping	−0.41	0.40	.31	-0.22	0.66	.74	−0.80	0.83	.33
Perceived scarcity # days without shopping	−0.04	0.29	.89	0.00	0.45	.99	−0.57	0.56	.31

a Each estimate was obtained from ordinary least squares regression. All regression models applied propensity score-matching weights, controlled for demographic variables, including gender, age, education, monthly income, and monthly expenditure, and used robust standard errors.

b To align with the timing of the lockdown, the analysis of “Days since quarantine” included only those who had quarantined (“During” group, n=326) or had not quarantined (“After” group, n=259) within 120 days.

c The analysis of “Days without shopping” was limited to individuals who had not shopped in the last 90 days for both the During (n=247) and After (n=124) groups.

**Table 3.** Effects of perceived scarcity and lockdown duration on time and risk preferences during and after the COVID-19 lockdown.^a^^b^,

Effect	Present bias (monetary)^c^			Present bias (health)^c^			Risk aversion (monetary)			Risk aversion (health)		
	b	SE	*P* value	b	SE	*P* value	b	SE	*P* value	b	se	*P* value
Panel A: During lockdown												
Perceived scarcity	1.05	0.84	.21	0.17	0.87	.85	0.32	0.65	.62	−0.03	0.52	.96
Days since quarantine	−4.46	4.48	.32	0.15	4.70	.97	−5.06	3.99	.20	−1.50	3.30	.65
Perceived scarcity # days since quarantine	0.34	1.34	.80	1.28	1.34	.34	4.68	1.44	<.001	−0.23	1.90	.90
Days without shopping	0.30	1.88	.87	−0.17	1.85	.93	−3.13	1.84	.09	−1.50	2.04	.46
Perceived scarcity # days without shopping	1.05	1.41	.46	0.77	1.39	.58	2.68	1.51	.08	−0.34	1.65	.84
Panel B: After lockdown												
Perceived scarcity	−0.42	0.32	.19	−0.15	0.32	.64	−0.00	0.24	.99	−0.13	0.26	.61
Days after quarantine	−6.00	3.40	.08	−9.38	3.73	.01	−0.19	1.99	.92	−3.44	1.93	.07
Perceived scarcity # days after quarantine	−3.80	2.07	.07	−6.24	2.28	.01	−1.20	1.23	.33	−2.67	1.11	.02
Days without shopping	−2.67	2.07	.20	−4.04	2.17	.06	−1.96	1.21	.10	−0.89	1.16	.44
Perceived scarcity # days without shopping	−2.70	1.37	.05	−3.02	1.42	.03	−1.55	0.83	.06	−1.08	0.81	.18

a Logistic regressions were performed for present bias, and ordinal regressions were performed for risk aversion.

b All regression models applied propensity score-matching weights, controlled for demographic variables, including gender, age, education, monthly income, and monthly expenditure, and used robust standard errors.

c The sample for Present bias was limited to participants ((“During” group, n=219; “After” group, n=226) who consistently chose either the earlier or later payment in both monetary and health time-discounting tasks, switching no more than once between options.

**Table 4.** Effects of perceived scarcity and lockdown duration on grocery, health, and temptation goods during and after the COVID-19 lockdown.^a^^b^

Effect	Proportion of groceries			Proportion of health items			Proportion of temptation goods		
	b	SE	*P* value	b	SE	*P* value	b	SE	*P* value
Panel A: During lockdown									
Perceived scarcity	0.67	0.27	.01	−0.61	0.26	.02	−0.29	0.29	.32
Days since quarantine	−4.43	1.41	<.001	3.98	1.46	.01	1.96	1.57	.21
Perceived scarcity # days since quarantine	1.52	0.54	.01	-1.41	0.42	<.001	−0.64	0.51	.21
Days without shopping	−1.01	0.79	.20	0.54	0.73	.46	0.75	0.72	.30
Perceived scarcity # days without shopping	0.77	0.59	.19	−0.64	0.57	.26	−0.38	0.53	.47
Panel B: After lockdown									
Perceived scarcity	−0.35	0.12	<.001	0.15	0.13	.24	0.29	0.12	.01
Days after quarantine	−1.82	1.01	.07	0.06	1.09	.96	2.10	1.06	.05
Perceived scarcity # days after quarantine	−1.07	0.57	.06	0.36	0.65	.58	0.97	0.60	.11
Days without shopping	0.05	0.55	.93	−0.02	0.44	.97	−0.04	0.69	.95
Perceived scarcity # days without shopping	−0.32	0.39	.41	0.16	0.30	.59	0.24	0.47	.61

a Each estimate was obtained from ordinary least squares regression.

b All regression models applied propensity score-matching weights, controlled for demographic variables, including gender, age, education, monthly income, and monthly expenditure, and used robust standard errors.

### Subgroup Analysis

**Figure 2.**
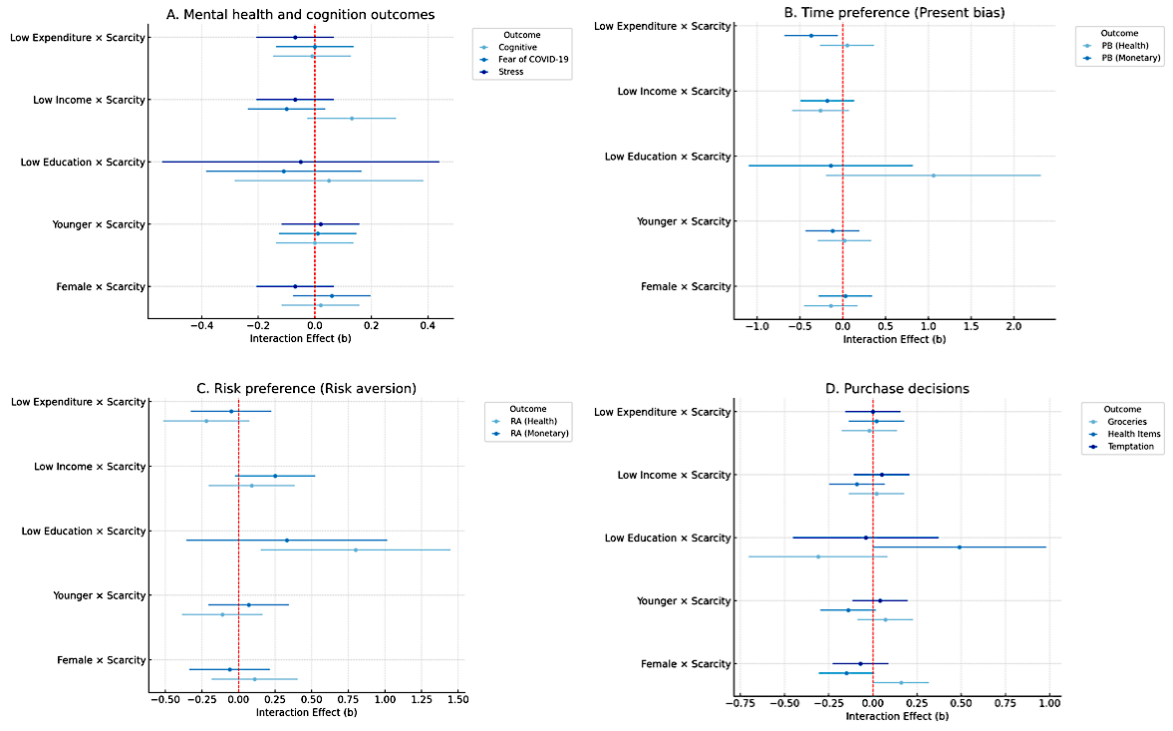
Subgroup analysis of interaction effects between perceived scarcity and demographic subgroups on perceived scarcity, stress, fear of COVID-19, time and risk preference, and spending trade-offs between grocery, health, and temptation. Estimates are plotted with 95% confidence intervals. Each estimate was obtained from ordinary least squares regression, except for present bias (logistic regression) and risk aversion (ordinal regression). All outcomes and continuous predictors were standardized (z-scores), and models adjusted for demographic covariates using propensity score-matching weights. Robust standard errors were used across all regressions.

Subgroup analyses revealed that the effect of perceived scarcity varied across demographic and economic groups (Figure 2). Women reallocated their budgets under scarcity by increasing grocery spending (standardized β coefficient=.16, *P*=.04) and reducing spending on health items (standardized β coefficient = –.15, *P*=.05). Low-education participants showed stronger protective behaviors: scarcity increased both their health-related risk aversion (standardized β coefficient =.80, *P*=.01) and health spending (standardized β coefficient =.49, *P*=.05). Interestingly, low-expenditure participants became less present-biased under scarcity (standardized β coefficient = –.37, *P*=.02), suggesting greater attentional focus on future orientation when facing resource constraints. In contrast, subgroup interactions with age and income showed no consistent or significant moderation effects. Full regression results for subgroup analysis are provided in Table S7 in Multimedia Appendix 2.

## Discussion

### Principal Findings

This study examined the psychological and behavioral impacts of perceived scarcity during and after the COVID-19 lockdown in Shanghai. By analyzing variables such as stress, fear of COVID-19, cognitive functioning, present bias, risk aversion, and budget allocation, the study aimed to understand how perceived scarcity arising from resource constraints influences mental health and decision-making processes during a pandemic. The findings provide empirical evidence on the nuanced effects of scarcity in a real-world crisis, advancing understanding of its impact on various psychological and behavioral outcomes.

Perceived scarcity and stress were significantly higher during the lockdown compared to after, aligning with existing literature that lockdown measures exacerbate mental health problems [6-8]. In both groups, individuals who perceived more scarcity reported higher stress, reinforcing prior research showing that poverty and resource constraints elevate stress levels [21,63]. The results extend this understanding by showcasing that this relationship holds true even in a relatively affluent, urban setting during a temporary crisis. The study highlighted a dose-response-type relationship: those who felt more scarcity had higher stress, a pattern also observed in other contexts of financial hardship and food insecurity [44]. The study found no significant increase in fear of COVID-19 during lockdown compared to after, while a significant negative interaction between perceived scarcity and days in quarantine was observed during lockdown. This may indicate that as the duration of home quarantine extends and individuals are less exposed to the pandemic environment, their perception of fear diminishes. The cognitive load theory offers one possible explanation for this link. In this case, many Shanghai residents faced difficulties obtaining groceries, leading to both cognitive (eg, time spent securing food) and emotional strain. While no significant group differences in cognitive functioning were found, interaction analyses revealed that longer quarantine was associated with lower cognitive performance during lockdown, and post-lockdown durations predicted improvement. These patterns align with longitudinal evidence on the cumulative effects of scarcity on cognition [30,64]. However, the cross-sectional design restricts the ability to infer a causal role of cognitive function in linking scarcity to stress and fear.

The stability of time preferences reported in both monetary- and health-framed choices is consistent with study [65] but contradicts study [66] conducted during COVID-19. Regarding the effects of perceived scarcity and lockdown duration, present bias was generally unchanged, except in health-framed choices post-lockdown, where individuals displayed a reduced present bias and became more future-oriented. However, the small sample sizes (n=91 for the During group; n=64 for the After group) in the analyses limit the confidence in these conclusions. Further research is necessary to confirm and clarify these trends. Risk preference in monetary-framed choices remained stable, aligning with previous studies [65,67,68]. In contrast, within health-framed choices, the During group exhibited greater risk aversion compared to the After group. This may be because the risk preference questions focused on vaccination, which was a more urgent and relevant health concern during the lockdown period. Furthermore, perceived scarcity, when interacting with days in quarantine, increased monetary risk aversion in the During group, while its interaction with days after quarantine decreased health-related risk aversion in the After group. Together, these findings indicate that perceived scarcity and the timing of lockdown influence individuals’ time and risk preferences differently across monetary and health-related contexts, highlighting the importance of context in understanding preferences and biases.

Lastly, when faced with trade-offs in purchasing decisions during the lockdown, participants prioritized spending on groceries over health items, reflecting immediate survival needs taking precedence under resource constraints [10,14,15]. This shift in spending priorities toward essential goods is also in line with findings from studies on purchase behaviors during COVID-19, such as online grocery shopping [12] and panic buying [11]. After lockdown, scarcity was associated with increased spending on temptation goods, indicating a shift toward non-essential purchases when restrictions eased. These changes highlight the dynamic nature of consumer behavior in response to external stressors and the role of scarcity in influencing trade-off decisions.

Our subgroup analyses also have important implications on the impacts of scarcity. Women, for instance, appeared to adjust their spending priorities to groceries under scarcity more than men. This could reflect gender roles in household provisioning, or possibly that women were willing to sacrifice personal or preventive health expenditures to ensure basic needs are met. Lower-educated participants showed stronger risk aversion to scarcity, which might indicate that individuals with less formal education react to scarcity by opting for safer choices, potentially neglecting larger or long-term benefits. This could be driven by a heightened perception of vulnerability. In contrast, neither age nor income level significantly moderated scarcity effects in our data, suggesting that individuals were affected similarly regardless of their age or economic status.

### Policy and Public Health Implications

The findings mentioned earlier offer handfuls of insights for policy makers and public health officials aiming to mitigate the negative consequences of perceived scarcity in crises. First, addressing the material needs and essential goods is paramount. Government should invest in resilient supply chains and emergency distribution networks, both online and offline, to facilitate access. For instance, establishing community distribution centers or partnering with grocery stores to deliver essentials to vulnerable populations can ensure that basic needs are met. Implementing real-time monitoring systems to track inventory levels and demand patterns can also help in proactive resource distribution. Using examples like Singapore’s successful governance, through food supply chain management, timely and transparent communication, and maintenance of public health service delivery, could help curb panic buying and reassure the public [69,70]. This study suggests that these proactive measures could greatly reduce perceived scarcity. If people believe essentials will remain available, they may experience less panic, less stress, and continue more rational decision patterns (eg, not overspending on groceries at the cost of medicine). Special attention should be given to subgroups who adjust their spending most under scarcity (eg, women managing household groceries) to ensure they have avenues for obtaining both food and health items so that critical needs (like medicines) are not sacrificed.

Second, findings suggest that mental health interventions may be necessary both during and after quarantine. Interventions aimed at enhancing coping strategies may be effective in reducing stress, anxiety, and fear during extended lockdowns [71]. This could involve providing virtual mental health services, such as online counseling or stress management workshops, to help individuals develop resilience [72]. To mitigate mental health challenges both during and after quarantine, governments should invest in digital mental health tools and offer support resources accessible to the public. Government can also partner with app developers to create mental health applications specifically designed for crisis situations, incorporating features such as daily wellness check-ins, stress management techniques, and mindfulness exercises [73]. Additionally, special attention should be given to vulnerable groups (eg, those who have lower income or lost income during the lockdown, people living alone, or those with pre-existing mental health issues) as they might experience compounded effects of scarcity and isolation. Proactive outreach and targeted interventions (such as providing remote counseling and peer support) for these groups could mitigate long-term mental health consequences. Our subgroup results also suggest tailoring support by demographic segment: for example, lower education individuals (who showed strong protective responses) may benefit from clear guidance to reinforce effective health behaviors without undue anxiety.

Third, public health communication plays a crucial role in managing perceived scarcity. Transparent updates on supply levels and distribution timelines can significantly reduce uncertainty-driven stress [74,75]. Public health campaigns should acknowledge inherent biases in individual decision-making and tailor messages accordingly. For instance, acknowledging that people become more risk-averse in health decisions during a pandemic (as observed in this study) could inform how to frame health communication messages. This could involve creating personalized messages that highlight potential health losses during times when risk aversion in health contexts is heightened. Post-lockdown, public health campaigns can promote balanced spending, encouraging individuals to maintain healthy consumption patterns. Another communications strategy is to combat misinformation that can exacerbate perceived scarcity (eg, rumors of food shortages). Engaging credible sources, community leaders, or even social media influencers to spread factual, calming information can prevent panic behaviors [76].

Finally, findings on spending trade-offs suggest a need for economic measures to support individuals under resource strain. If people are cutting back on health-related spending to afford groceries, governments or NGOs could introduce programs like vouchers for health supplies or food assistance to remove the dilemma of choosing between food and medicine. Post-crisis, stimulating the economy in a way that encourages a balanced return to normal consumption, perhaps through incentives for purchasing health-related goods or campaigns to promote healthy lifestyles, could be beneficial. In summary, policy interventions should tackle both the objective and subjective aspects of scarcity: ensuring actual availability of resources and concurrently addressing the psychology of scarcity by offering support and communication that reduce uncertainty and stress.

### Limitations and Future Research

This study has limitations that suggest avenues for future research. First, the cross-sectional design, comparing 2 independent groups, limits causal inference or change tracking within individuals over time. This study leveraged a quasi-natural experimental design, which limited the ability to examine certain assumptions (eg, parallel trends in outcomes prior to the lockdown) that would strengthen causal claims. Future studies could use a longitudinal design, tracking the same participants across different stages of a crisis, which would offer insights into how perceived scarcity impacts individuals over time. Second, it is important to acknowledge the sampling method in the given context of a period of lockdown in a single city area may not fully capture the representativeness of the population (all adults in Shanghai during the lockdown) and fall short in addressing the inherent uncertainty in generalizing these results to other populations. The inference in this study is bounded by the specific population and sampling conditions of this study. Furthermore, the reliance on self-reported data introduces potential biases, such as recall bias or social desirability bias. Future research could incorporate objective data, such as actual purchasing behaviors through transaction records or physiological stress indicators like cortisol levels. Additionally, the use of incentivized tasks for measuring time and risk preferences could provide more accurate assessments of decision-making behaviors. Another limitation is the limited sample size for specific analyses, particularly those related to present bias, which may affect result reliability. Future studies should aim for larger samples to confirm these findings and enhance statistical power. Broadening participants from different regions and cultural backgrounds can also help generalize the findings and explore cultural influences on the effects of scarcity. Finally, this research highlights the need to further examine how perceived scarcity shapes mental health and decision-making in the long term, especially as crises like pandemics and climate-related events may become more frequent. Future research could investigate the potential for lasting behavioral changes induced by scarcity, such as persistent shifts in spending priorities and purchase behaviors.

### Conclusions

This paper provides a nuanced understanding of the psychological and behavioral impacts of perceived scarcity during a pandemic, focusing on an urban population during and after the COVID-19 lockdown in Shanghai. The findings reveal that scarcity significantly influences stress levels, cognitive function, financial risk aversion, and budget allocation, while fear and present bias show more moderate effects. From a theoretical perspective, the study extends the concept of perceived scarcity to encompass situational constraints experienced by higher income populations during crises and advances current literature by demonstrating that the psychological and behavioral effects of perceived scarcity are multifaceted and context dependent. Therefore, this study advances theoretical understanding by highlighting the domain-specific effects of perceived scarcity and the resilience observed in cognitive functioning among highly educated populations. From a policy perspective, findings provide evidence for policy makers to design interventions that address both the material and psychological needs of populations during times of scarcity. By implementing targeted strategies, such as leveraging technology for resource management, providing mental health support, and tailoring communication to individual biases, governments can mitigate the negative impacts of scarcity and enhance resilience in future crises.

## Supplementary material

10.2196/69496 Multimedia Appendix 1 Web-based survey questions.

10.2196/69496 Multimedia Appendix 2 Supplementary tables.

## Abbreviations

OLS: ordinary least squares
PSM: propensity score matching
PSS: Perceived Stress Scale
VIF: variance inflation factor

## References

[1] Flaxman S, Mishra S, Gandy A (2020). Estimating the effects of non-pharmaceutical interventions on COVID-19 in Europe. Nature New Biol.

[2] Liu Y, Wang W, Wong WK, Zhu W (2024). Effectiveness of non-pharmaceutical interventions for COVID-19 in USA. Sci Rep.

[3] Yang M, He Z, Zhang Y, Liu T, Ming WK (2024). Pandemic fatigue and preferences for COVID-19 public health and social measures in China: nationwide discrete choice experiment. JMIR Public Health Surveill.

[4] Sugaya N, Yamamoto T, Suzuki N, Uchiumi C (2024). Loneliness and social isolation factors under the prolonged COVID-19 pandemic in Japan: 2-year longitudinal study. JMIR Public Health Surveill.

[5] Lei L, Huang X, Zhang S, Yang J, Yang L, Xu M (2020). Comparison of prevalence and associated factors of anxiety and depression among people affected by versus people unaffected by quarantine during the COVID-19 epidemic in Southwestern China. Med Sci Monit.

[6] Serrano-Alarcón M, Kentikelenis A, Mckee M, Stuckler D (2022). Impact of COVID-19 lockdowns on mental health: evidence from a quasi-natural experiment in England and Scotland. Health Econ.

[7] Pierce M, Hope H, Ford T (2020). Mental health before and during the COVID-19 pandemic: a longitudinal probability sample survey of the UK population. Lancet Psychiatry.

[8] Robinson E, Sutin AR, Daly M, Jones A (2022). A systematic review and meta-analysis of longitudinal cohort studies comparing mental health before versus during the COVID-19 pandemic in 2020. J Affect Disord.

[9] Carroll N, Sadowski A, Laila A (2020). The impact of COVID-19 on health behavior, stress, financial and food security among middle to high income canadian families with young children. Nutrients.

[10] Posel D, Oyenubi A, Kollamparambil U (2021). Job loss and mental health during the COVID-19 lockdown: evidence from South Africa. PLoS ONE.

[11] Yuen KF, Tan LS, Wong YD, Wang X (2022). Social determinants of panic buying behaviour amidst COVID-19 pandemic: the role of perceived scarcity and anticipated regret. Journal of Retailing and Consumer Services.

[12] Budziński W, Daziano R (2023). Preferences for online grocery shopping during the COVID-19 pandemic - the role of fear-related attitudes. J Choice Model.

[13] Schurer S, Atalay K, Glozier N, Vera-Toscano E, Wooden M (2023). Quantifying the human impact of Melbourne’s 111-day hard lockdown experiment on the adult population. Nat Hum Behav.

[14] Harling G, Gómez-Olivé FX, Tlouyamma J (2021). Protective behaviors and secondary harms resulting from nonpharmaceutical interventions during the COVID-19 epidemic in South Africa: multisite, prospective longitudinal study. JMIR Public Health Surveill.

[15] Liao Q, Xiao J, Cheung J (2021). Community psychological and behavioural responses to coronavirus disease 2019 over one year of the pandemic in 2020 in Hong Kong. Sci Rep.

[16] Prati G, Mancini AD (2021). The psychological impact of COVID-19 pandemic lockdowns: a review and meta-analysis of longitudinal studies and natural experiments. Psychol Med.

[17] O’Connor RC, Wetherall K, Cleare S (2021). Mental health and well-being during the COVID-19 pandemic: longitudinal analyses of adults in the UK COVID-19 Mental Health & Wellbeing study. Br J Psychiatry.

[18] Chan CP, Lee SS, Kwan TH, Wong SYS, Yeoh EK, Wong NS (2024). Population behavior changes underlying phasic shifts of SARS-CoV-2 exposure settings across 3 omicron epidemic waves in Hong Kong: prospective cohort study. JMIR Public Health Surveill.

[19] de Vocht F, Katikireddi SV, McQuire C, Tilling K, Hickman M, Craig P (2021). Conceptualising natural and quasi experiments in public health. BMC Med Res Methodol.

[20] Campbell DT, Stanley JC (2015). Experimental and Quasi-Experimental Designs for Research.

[21] Haushofer J, Fehr E (2014). On the psychology of poverty. Science.

[22] de Bruijn EJ, Antonides G (2022). Poverty and economic decision making: a review of scarcity theory. Theory Decis.

[23] Adamkovič M, Martončik M (2017). A review of consequences of poverty on economic decision-making: a hypothesized model of a cognitive mechanism. Front Psychol.

[24] Mani A, Mullainathan S, Shafir E, Zhao J (2013). Poverty impedes cognitive function. Science.

[25] Goldsmith K, Griskevicius V, Hamilton R (2020). Scarcity and consumer decision making: is scarcity a mindset, a threat, a reference point, or a journey?. Journal of the Association for Consumer Research.

[26] Shah AK, Zhao J, Mullainathan S, Shafir E (2018). Money in the mental lives of the poor. Soc Cogn.

[27] Hamilton R, Thompson D, Bone S (2019). The effects of scarcity on consumer decision journeys. J of the Acad Mark Sci.

[28] McGuire J, Kaiser C, Bach-Mortensen AM (2022). A systematic review and meta-analysis of the impact of cash transfers on subjective well-being and mental health in low- and middle-income countries. Nat Hum Behav.

[29] Ridley M, Rao G, Schilbach F, Patel V (2020). Poverty, depression, and anxiety: causal evidence and mechanisms. Science.

[30] Lichand G, Mani A (2020). Cognitive droughts. SSRN Journal.

[31] Mertens G, Lodder P, Smeets T, Duijndam S (2023). Pandemic panic? Results of a 14-month longitudinal study on fear of COVID-19. J Affect Disord.

[32] Luo F, Ghanei Gheshlagh R, Dalvand S, Saedmoucheshi S, Li Q (2021). Systematic review and meta-analysis of fear of COVID-19. Front Psychol.

[33] Pfeifer LS, Heyers K, Ocklenburg S, Wolf OT (2021). Stress research during the COVID-19 pandemic and beyond. Neurosci Biobehav Rev.

[34] Kowal M, Coll-Martín T, Ikizer G (2020). Who is the most stressed during the COVID-19 pandemic? Data from 26 countries and areas. Appl Psychol Health Well Being.

[35] Demont T, Horta-Sáenz D, Raiber E (2024). Exposure to worrisome topics can increase cognitive performance when incentivized by a performance goal. Sci Rep.

[36] Tversky A, Kahneman D (1974). Judgment under uncertainty: heuristics and biases. Science.

[37] Frederick S, Loewenstein G, O’donoghue T (2002). Time discounting and time preference: a critical review. J Econ Lit.

[38] O’Donoghue T, Rabin M (1999). Doing it now or later. American Economic Review.

[39] Laibson D (1997). Golden eggs and hyperbolic discounting. Q J Econ.

[40] Kahneman D, Tversky A (2013). Handbook of the Fundamentals of Financial Decision Making: Part I World Scientific.

[41] Harrison GW, Lau MI, Rutström EE (2010). Individual discount rates and smoking: evidence from a field experiment in Denmark. J Health Econ.

[42] Ida T (2014). A quasi-hyperbolic discounting approach to smoking behavior. Health Econ Rev.

[43] Ikeda S, Kang MI, Ohtake F (2016). Behavioral Economics of Preferences, Choices, and Happiness.

[44] Dalton PS, Nhung N, Rüschenpöhler J (2020). Worries of the poor: the impact of financial burden on the risk attitudes of micro-entrepreneurs. J Econ Psychol.

[45] Bartoš V, Bauer M, Chytilová J, Levely I (2021). Psychological effects of poverty on time preferences. The Economic Journal.

[46] Carvalho LS, Meier S, Wang SW (2016). Poverty and economic decision-making: evidence from changes in financial resources at Payday. Am Econ Rev.

[47] Burlacu S, Mani A, Ronzani P, Savadori L (2023). The preoccupied parent: how financial concerns affect child investment choices. J Behav Exp Econ.

[48] Lichand G, Bettinger E, Cunha N, Madeira R (2020). The psychological effects of poverty on investments in children’s human capital. SSRN Journal.

[49] Burlacu S, Kažemekaitytė A, Ronzani P, Savadori L (2022). Blinded by worries: sin taxes and demand for temptation under financial worries. Theory Decis.

[50] Park YR, Ho Y, Hallez K, Kaur S, Srinivasan M, Zhao J (2025). The psychology of poverty: current and future directions. Curr Dir Psychol Sci.

[51] Craig P, Cooper C, Gunnell D (2012). Using natural experiments to evaluate population health interventions: new Medical Research Council guidance. J Epidemiol Community Health.

[52] Craig P, Katikireddi SV, Leyland A, Popham F (2017). Natural experiments: an overview of methods, approaches, and contributions to public health intervention research. Annu Rev Public Health.

[53] Dunning T (2012). Natural Experiments in the Social Sciences: A Design-Based Approach.

[54] William K (2018). China market research tool. Medium.

[55] Byun SE, Sternquist B (2008). The antecedents of in-store hoarding: measurement and application in the fast fashion retail environment. The International Review of Retail, Distribution and Consumer Research.

[56] Cohen S, Kamarck T, Mermelstein R (1983). A global measure of perceived stress. J Health Soc Behav.

[57] Ahorsu DK, Lin CY, Imani V, Saffari M, Griffiths MD, Pakpour AH (2022). The fear of COVID-19 scale: development and initial validation. Int J Ment Health Addict.

[58] Raven J (2000). The Raven’s progressive matrices: change and stability over culture and time. Cogn Psychol.

[59] Schilbach F, Schofield H, Mullainathan S (2016). The psychological lives of the poor. Am Econ Rev.

[60] Eckel CC, Grossman PJ (2002). Sex differences and statistical stereotyping in attitudes toward financial risk. Evol Hum Behav.

[61] Tversky A, Kahneman D (1981). The framing of decisions and the psychology of choice. Science.

[62] Fehr D, Fink G, Jack BK (2022). Poor and rational: decision-making under scarcity. Journal of Political Economy.

[63] Haushofer J, Shapiro J (2016). The short-term impact of unconditional cash transfers to the poor: experimental evidence from Kenya. Q J Econ.

[64] Mani A, Mullainathan S, Shafir E, Zhao J (2020). Scarcity and cognitive function around payday: a conceptual and empirical analysis. Journal of the Association for Consumer Research.

[65] Drichoutis AC, Nayga RM (2022). On the stability of risk and time preferences amid the COVID-19 pandemic. Exp Econ.

[66] Li KK, Huang B, Tam T, Hong Y yi (2020). Does the COVID-19 pandemic affect people’s social and economic preferences? Evidence from China. SSRN Journal.

[67] Zhang P, Palma MA (2021). Stability of risk preferences during COVID-19: evidence from four measurements. Front Psychol.

[68] Angrisani M, Cipriani M, Guarino A, Kendall R, Ortiz de Zarate J (2020). Risk preferences at the time of COVID-19: an experiment with professional traders and students. SSRN Journal.

[69] Tortajada C, Lim NSW (2021). Food security and COVID-19: impacts and resilience in Singapore. Front Sustain Food Syst.

[70] Chua AQ, Tan MMJ, Verma M (2020). Health system resilience in managing the COVID-19 pandemic: lessons from Singapore. BMJ Glob Health.

[71] Chen L, Chu Q, Xu C (2024). Psychological responses and factors associated with depression and anxiety in entry personnel under quarantine during pandemic in China. Front Public Health.

[72] Escobar-Agreda S, Silva-Valencia J, Soto-Becerra P (2024). Patient engagement with and perceptions of the COVIDA project, a volunteer-led telemonitoring and teleorientation service for COVID-19 community management: mixed methods study. JMIR Form Res.

[73] Maloney S, Montero-Marin J, Kuyken W (2024). Mindfulness-based cognitive therapy-taking it further (MBCT-TiF) compared to ongoing mindfulness practice (OMP) in the promotion of well-being and mental health: a randomised controlled trial with graduates of MBCT and MBSR. Behav Res Ther.

[74] Ho SS, Chuah ASF, Ho VS, Rosenthal S, Kim HK, Soh SSH (2024). Crisis and emergency risk communication and emotional appeals in COVID-19 public health messaging: quantitative content analysis. J Med Internet Res.

[75] Kwak M, Kim BJ, Chung JB (2024). Serious game development for public health: participatory design approach to COVID-19 quarantine policy education. JMIR Serious Games.

[76] Hermann E (2022). Leveraging artificial intelligence in marketing for social good-an ethical perspective. J Bus Ethics.

[77] OSF home. Effects of perceived scarcity on mental health, cognitive functioning, time and risk preferences and decision-making during and after covid-19 lockdown: a quasi-natural experimental study.

