# Supplementary material for: Effects of Perceived Scarcity on Mental Health, Time and Risk Preferences, and Decision-Making During and After COVID-19 Lockdown: Quasi-Natural Experimental Study
Source: JMIR Public Health Surveill. 2025 Aug 29;11:e69496. doi: 10.2196/69496 (PMC12421341; doi:10.2196/69496)
Supplement: Multimedia Appendix 1 [file publichealth-v11-e69496-s001.docx]

**Multimedia Appendix 1**

**Online survey questions**

**1. The questions in this scale ask you about your feelings and thoughts during the last month. In each case, you will be asked to indicate by circling how often you felt or thought a certain way.**

|  | never | almost never | sometimes | fairly often | very often |
| --- | --- | --- | --- | --- | --- |
| 1.1 In the last month, how often have you been upset because of something that happened unexpectedly? | ○ | ○ | ○ | ○ | ○ |
| 1.2 In the last month, how often have you felt that you were unable to control the important things in your life? | ○ | ○ | ○ | ○ | ○ |
| 1.3 In the last month, how often have you felt nervous and “stressed”? | ○ | ○ | ○ | ○ | ○ |
| 1.4 In the last month, how often have you felt confident about your ability to handle your personal problems? | ○ | ○ | ○ | ○ | ○ |
| 1.5 In the last month, how often have you felt that things were going your way? | ○ | ○ | ○ | ○ | ○ |
| 1.6 In the last month, how often have you found that you could not cope with all the things that you had to do? | ○ | ○ | ○ | ○ | ○ |
| 1.7 In the last month, how often have you been able to control irritations in your life? | ○ | ○ | ○ | ○ | ○ |
| 1.8 In the last month, how often have you felt that you were on top of things? | ○ | ○ | ○ | ○ | ○ |
| 1.9 In the last month, how often have you been angered because of things that were outside of your control? | ○ | ○ | ○ | ○ | ○ |
| 1.10 In the last month, how often have you felt difficulties were piling up so high that you could not overcome them? | ○ | ○ | ○ | ○ | ○ |

**2. The descriptions in this scale ask you about your feelings and thoughts during the last month. In each case, you will be asked to indicate by circling how much you agree or disagree with the description.**

2.1 In the last month, the products that I wanted to buy were very limited.

○ Totally disagree ○2 ○3 ○4 ○5 ○6 ○7 ○8 ○9 ○ Totally agree

2.2 In the last month, the brand availability for a product was very limited.

○ Totally disagree ○2 ○3 ○4 ○5 ○6 ○7 ○8 ○9 ○ Totally agree

2.3 In the last month, the sizes of a product were very limited.

○ Totally disagree ○2 ○3 ○4 ○5 ○6 ○7 ○8 ○9 ○ Totally agree

2.4 In the last month, the types of products were very limited.

○ Totally disagree ○2 ○3 ○4 ○5 ○6 ○7 ○8 ○9 ○ Totally agree

**3. The descriptions in this scale (1-10) ask you about your feelings and thoughts during the last month. In each case, you will be asked to indicate by circling how much you agree or disagree with the description.**

3.1 I am very afraid of covid-19.

○ Totally disagree ○2 ○3 ○4 ○5 ○6 ○7 ○8 ○9 ○ Totally agree

3.2 It makes me uncomfortable to think about covid-19.

○ Totally disagree ○2 ○3 ○4 ○5 ○6 ○7 ○8 ○9 ○ Totally agree

3.3 My hands become clammy when I think about covid-19.

○ Totally disagree ○2 ○3 ○4 ○5 ○6 ○7 ○8 ○9 ○ Totally agree

3.4 I am afraid of losing my life because of covid-19.

○ Totally disagree ○2 ○3 ○4 ○5 ○6 ○7 ○8 ○9 ○ Totally agree

3.5 When watching news and stories about covid-19 on social media, I become nervous or anxious.

○ Totally disagree ○2 ○3 ○4 ○5 ○6 ○7 ○8 ○9 ○ Totally agree

3.6 I cannot sleep because I’m worrying about getting covid-19.

○ Totally disagree ○2 ○3 ○4 ○5 ○6 ○7 ○8 ○9 ○ Totally agree

3.7 My heart races or palpitates when I think about getting covid-19.

○ Totally disagree ○2 ○3 ○4 ○5 ○6 ○7 ○8 ○9 ○ Totally agree

**4. Please select the appropriate pattern to fill in the vacancy according to the rules of the symbols or patterns in the big picture, and select the numbering option corresponding to the pattern.**

4.1


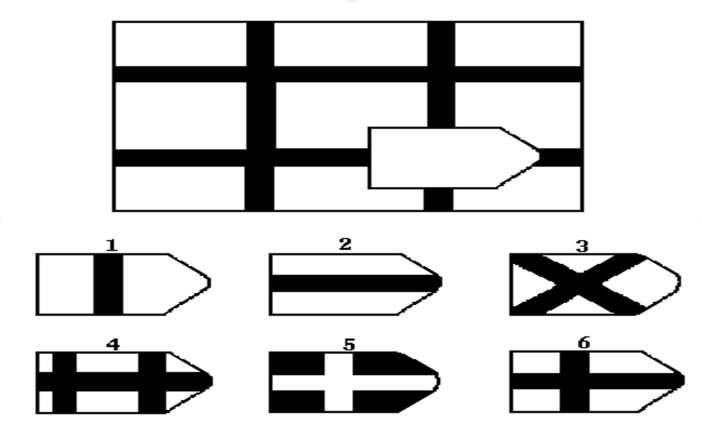


4.2


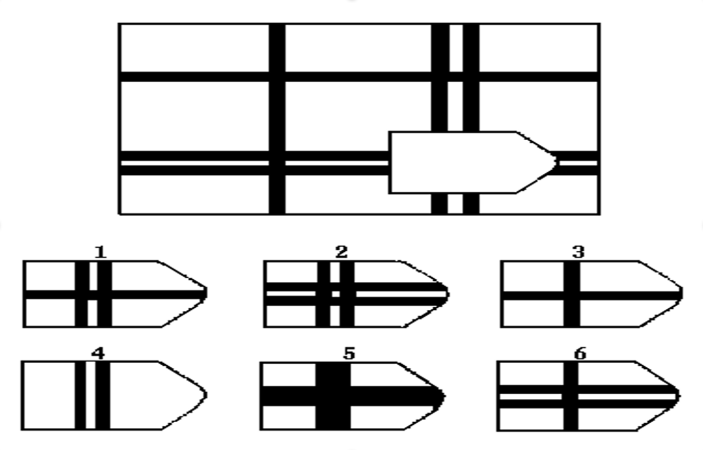


4.3

 
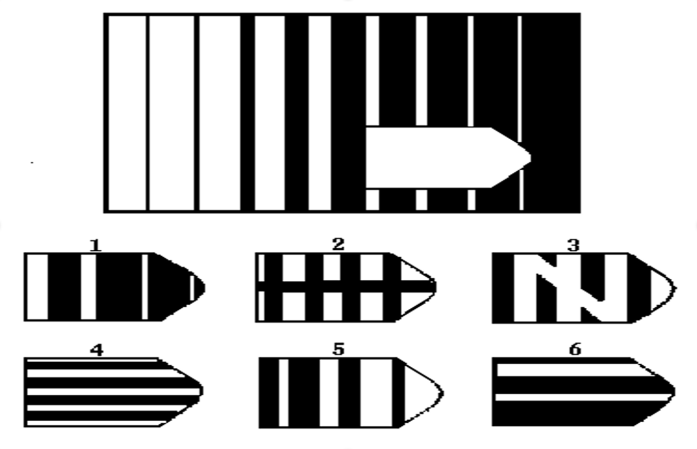


4.4


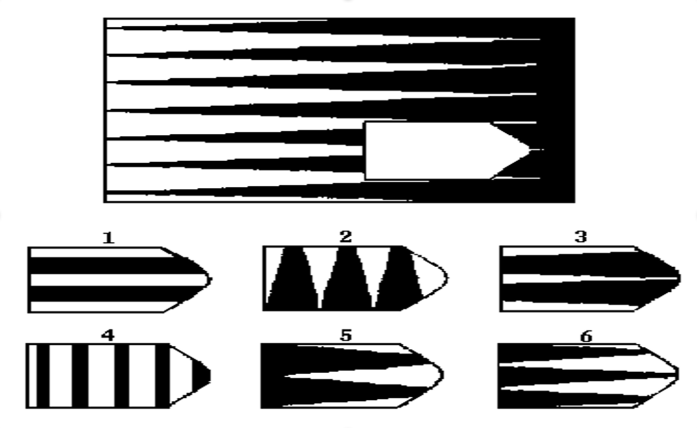


4.5


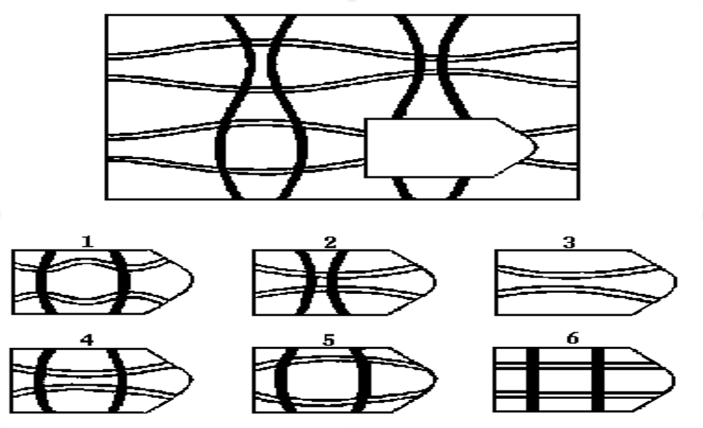


4.6


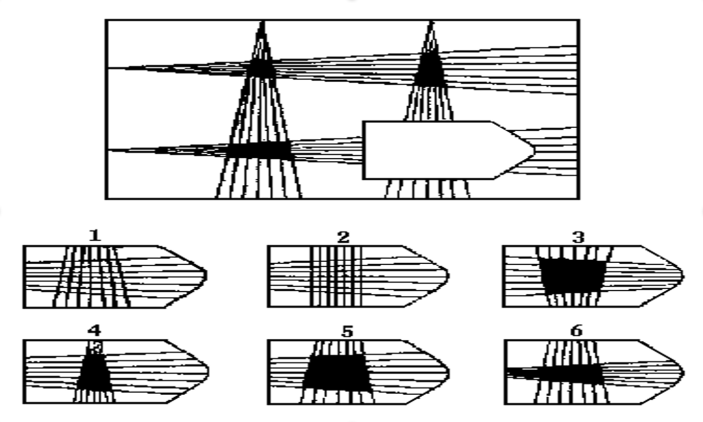


4.7

 
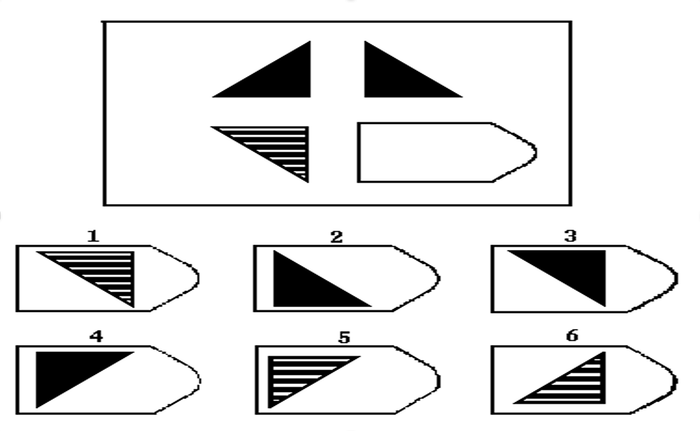


4.8

 
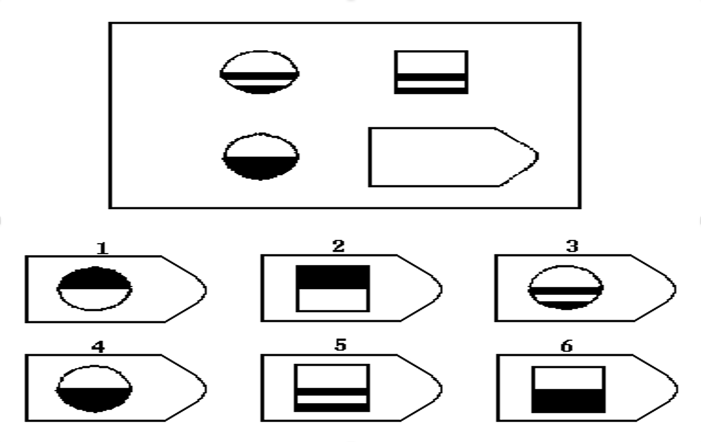


4.9

 
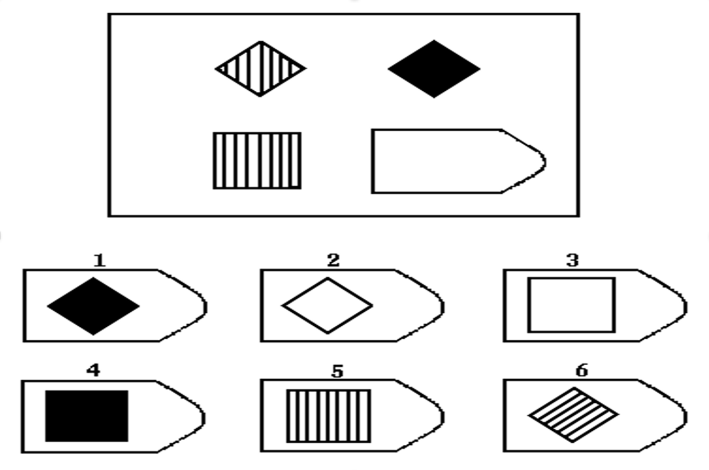


4.10

 
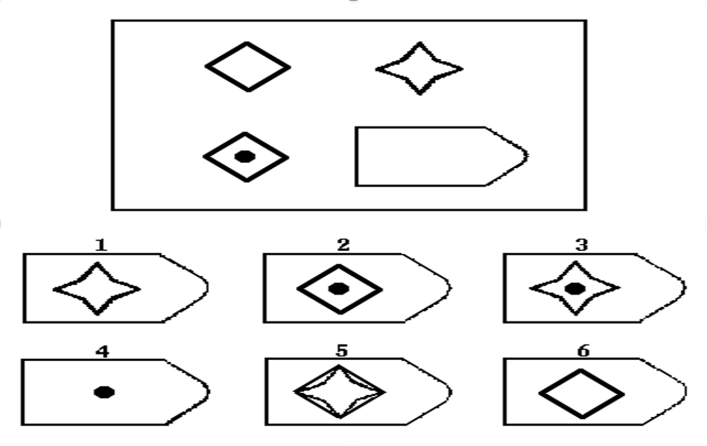


4.11

 
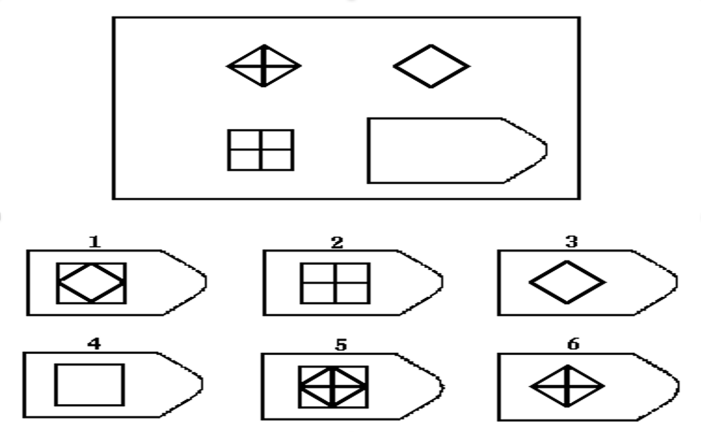


4.12

 
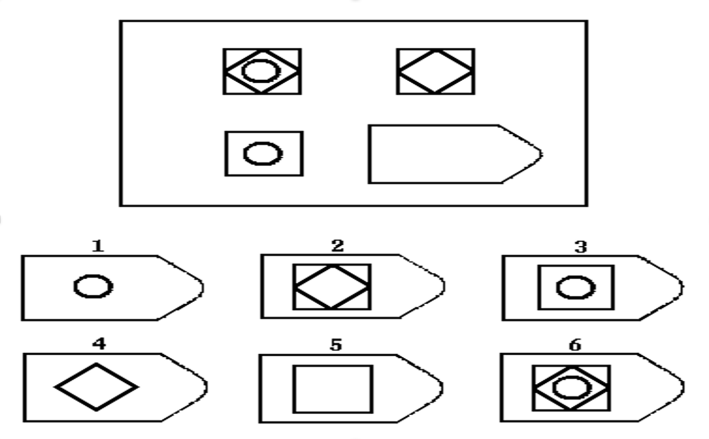


**5. Risk choice scenarios
Scenario 1: The following are 6 groups of lottery games. Each group of lottery contains 2 winning amounts, and the odds of winning are 50%.**

Please choose which group of lottery games you prefer to play according to your own preferences:

| ○ lottery 1: 50% chance winning ¥280，50% chance winning ¥280 |
| --- |
| ○ lottery 2: 50% chance winning ¥360，50% chance winning ¥240 |
| ○ lottery 3: 50% chance winning ¥440，50% chance winning ¥200 |
| ○ lottery 4: 50% chance winning ¥520，50% chance winning ¥160 |
| ○ lottery 5: 50% chance winning ¥600，50% chance winning ¥120 |
| ○ lottery 6: 50% chance winning ¥700，50% chance winning ¥20 |

**Scenario 2: Imagine that there is an outbreak of an unusual disease. There are currently 6 vaccine products to choose from. Each vaccine will produce 2 possible immunity durations after vaccination, and the probability of occurrence is 50%.**

Please select which vaccine you would prefer:

| ○ vaccine 1：50% chance immunity for 6 months，50% chance immunity for 6 months |
| --- |
| ○ vaccine 2：50% chance immunity for 8 months，50% chance immunity for 5 months |
| ○ vaccine 3：50% chance immunity for 10 months，50% chance immunity for 4 months |
| ○ vaccine 4：50% chance immunity for 12 months，50% chance immunity for 3 months |
| ○ vaccine 5：50% chance immunity for 14 months，50% chance immunity for 2 months |
| ○ vaccine 6：50% chance immunity for 17 months，50% chance immunity for 15 days |

**Scenario 3: Imagine that there is an outbreak of an unusual disease, which is expected to kill 600 people. Two alternative programs to combat the disease have been proposed. Assume that the exact scientific estimate of the consequences of the programs are framed as follows.**

Please indicate which of the two programs would you favor (Frame 1):

| ○ If program A is adopted, 200 people will be saved. |
| --- |
| ○ If program B is adopted, there is a 1in 3 chance that 600 people will be saved, and a 2 in 3 chance that no people will be saved. |

Please indicate which of the two programs would you favor (Frame 2):

| ○ If program A is adopted, 400 people will die. |
| --- |
| ○ If program B is adopted, there is a 1in 3 chance that nobody will be die, and a 2 in 3 chance that 600 people will die. |

**6. Time choice scenarios

Scenario 1: Imagine that after completing a job, you have two ways of getting paid:
A. to receive a smaller amount of payment earlier
B. to receive a larger amount of payment later**

In the following two sets of decisions, please make the choice in which of the two ways you would like to receive the payment:

| First set (payment today vs. 1 month) | A | B |
| --- | --- | --- |
| Decision 1: A. ¥980 today B. ¥1000 in 1 month | ○ | ○ |
| Decision 2: A. ¥960 today B. ¥1000 in 1 month | ○ | ○ |
| Decision 3: A. ¥920 today B. ¥1000 in 1 month | ○ | ○ |
| Decision 4: A. ¥880 today B. ¥1000 in 1 month | ○ | ○ |
| Decision 5: A. ¥820 today B. ¥1000 in 1 month | ○ | ○ |
| Decision 6: A. ¥760 today B. ¥1000 in 1 month | ○ | ○ |
| Decision 7: A. ¥680 today B. ¥1000 in 1 month | ○ | ○ |
| Decision 8: A. ¥600 today B. ¥1000 in 1 month | ○ | ○ |

| Second set (payment in 6 months vs. 7 months) | A | B |
| --- | --- | --- |
| Decision 1: A. ¥980 in 6 months B. ¥1000 in 7 months | ○ | ○ |
| Decision 2: A. ¥960 in 6 months B. ¥1000 in 7 months | ○ | ○ |
| Decision 3: A. ¥920 in 6 months B. ¥1000 in 7 months | ○ | ○ |
| Decision 4: A. ¥880 in 6 months B. ¥1000 in 7 months | ○ | ○ |
| Decision 5: A. ¥820 in 6 months B. ¥1000 in 7 months | ○ | ○ |
| Decision 6: A. ¥760 in 6 months B. ¥1000 in 7 months | ○ | ○ |
| Decision 7: A. ¥680 in 6 months B. ¥1000 in 7 months | ○ | ○ |
| Decision 8: A. ¥600 in 6 months B. ¥1000 in 7 months | ○ | ○ |

**Scenario 2: Imagine that you've been quite busy recently, but your headache continues to bother you. If you go to see the doctor right away, it may delay your work and lose job payment; if you go in one month, the headache may worsen.**

Considering mainly time and payment loss, please make the choice when you will go to see the doctor in the following set of decisions:

|  | A | B |
| --- | --- | --- |
| Decision 1: A. see doctor today, loss ¥600  B. see doctor in 1 month, loss ¥600 | ○ | ○ |
| Decision 2: A. see doctor today, loss ¥680  B. see doctor in 1 month, loss ¥600 | ○ | ○ |
| Decision 3: A. see doctor today, loss ¥760  B. see doctor in 1 month, loss ¥600 | ○ | ○ |
| Decision 4: A. see doctor today, loss ¥820  B. see doctor in 1 month, loss ¥600 | ○ | ○ |
| Decision 5: A. see doctor today, loss ¥880  B. see doctor in 1 month, loss ¥600 | ○ | ○ |
| Decision 6: A. see doctor today, loss ¥920  B. see doctor in 1 month, loss ¥600 | ○ | ○ |
| Decision 7: A. see doctor today, loss ¥960  B. see doctor in 1 month, loss ¥600 | ○ | ○ |
| Decision 8: A. see doctor today, loss ¥980  B. see doctor in 1 month, loss ¥600 | ○ | ○ |

Considering mainly time and travel distance, please make the choice when you will go to see the doctor in the following set of decisions:

|  | A | B |
| --- | --- | --- |
| Decision 1: A. see doctor today, travel distance 3km  B. see doctor in one month, travel distance 3km | ○ | ○ |
| Decision 2: A. see doctor today, travel distance 5km  B. see doctor in one month, travel distance 3km | ○ | ○ |
| Decision 3: A. see doctor today, travel distance 7km  B. see doctor in one month, travel distance 3km | ○ | ○ |
| Decision 4: A. see doctor today, travel distance 9km  B. see doctor in one month, travel distance 3km | ○ | ○ |
| Decision 5: A. see doctor today, travel distance 11km  B. see doctor in one month, travel distance 3km | ○ | ○ |
| Decision 6: A. see doctor today, travel distance 13km  B. see doctor in one month, travel distance 3km | ○ | ○ |
| Decision 7: A. see doctor today, travel distance 15km  B. see doctor in one month, travel distance 3km | ○ | ○ |
| Decision 8: A. see doctor today, travel distance 17km  B. see doctor in one month, travel distance 3km | ○ | ○ |

**Scenario 3:** **A physical examination bundle costs around 1,000 yuan. Suppose there is a subsidy policy, and you have two ways of receiving the subsidy:**

**A. to receive a smaller amount of subsidy earlier
B. to receive a larger amount of subsidy later**

In the following two sets of decisions, please make the choice in which of the two ways you would like to receive the subsidy:

| First set (subsidy today vs. 1 month) | A | B |
| --- | --- | --- |
| Decision 1: A. physical exam today, subsidy ¥980  B. physical exam in 1 month, subsidy ¥1000 | ○ | ○ |
| Decision 2: A. physical exam today, subsidy ¥960  B. physical exam in 1 month, subsidy ¥1000 | ○ | ○ |
| Decision 3: A. physical exam today, subsidy ¥920  B. physical exam in 1 month, subsidy ¥1000 | ○ | ○ |
| Decision 4: A. physical exam today, subsidy ¥880  B. physical exam in 1 month, subsidy ¥1000 | ○ | ○ |
| Decision 5: A. physical exam today, subsidy ¥820  B. physical exam in 1 month, subsidy ¥1000 | ○ | ○ |
| Decision 6: A. physical exam today, subsidy ¥760  B. physical exam in 1 month, subsidy ¥1000 | ○ | ○ |
| Decision 7: A. physical exam today, subsidy ¥680  B. physical exam in 1 month, subsidy ¥1000 | ○ | ○ |
| Decision 8: A. physical exam today, subsidy ¥600  B. physical exam in 1 month, subsidy ¥1000 | ○ | ○ |

| Second set (subsidy in 6 months vs. 7 months) | A | B |
| --- | --- | --- |
| Decision 1: A. physical exam in 6 months, subsidy ¥980  B. physical exam in 7 months, subsidy ¥1000 | ○ | ○ |
| Decision 2: A. physical exam in 6 months, subsidy ¥960  B. physical exam in 7 months, subsidy ¥1000 | ○ | ○ |
| Decision 3: A. physical exam in 6 months, subsidy ¥920  B. physical exam in 7 months, subsidy ¥1000 | ○ | ○ |
| Decision 4: A. physical exam in 6 months, subsidy ¥880  B. physical exam in 7 months, subsidy ¥1000 | ○ | ○ |
| Decision 5: A. physical exam in 6 months, subsidy ¥820  B. physical exam in 7 months, subsidy ¥1000 | ○ | ○ |
| Decision 6: A. physical exam in 6 months, subsidy ¥760  B. physical exam in 7 months, subsidy ¥1000 | ○ | ○ |
| Decision 7: A. physical exam in 6 months, subsidy ¥680  B. physical exam in 7 months, subsidy ¥1000 | ○ | ○ |
| Decision 8: A. physical exam in 6 months, subsidy ¥600  B. physical exam in 7 months, subsidy ¥1000 | ○ | ○ |

**7. Budget allocation

Scenario: In the following task you are asked to choose what goods to purchase with a budget of ¥300. You will see a list of available goods, with a picture, title and the price displayed for each of them. By clicking on the ‘+’ button, the goods will be added to the shopping cart. Try to spend as close to the ¥300 budget as possible.**

| 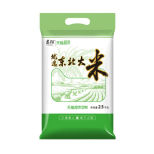 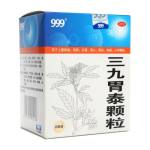 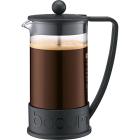 □ rice __¥19.8__ □ Weitai granule ___¥21.6__ □ Coffee pot __¥59_____ |
| --- |
| 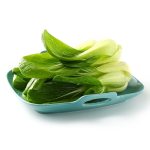 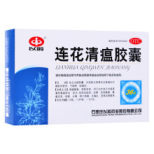 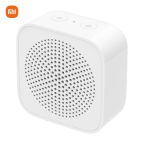 □ Chinese leaf _¥11.3_ □ Lianhua Qingwen capsules _¥21.6_ □ Bluetooth soundbox __¥49__ |
| 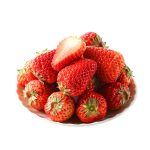 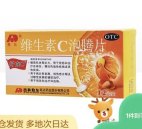 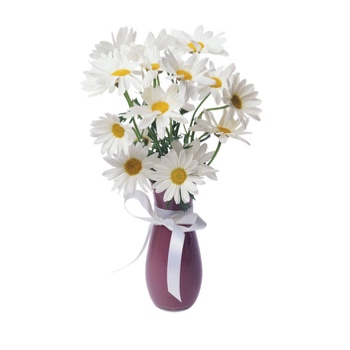  □ Strawberry __¥23.5__ □ Vitamin C __¥19.8__ □ Fresh flowers __¥19.8__ |
| 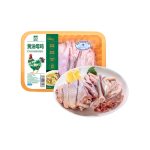 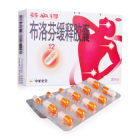 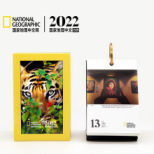 □ Chicken __¥36.8_____ □ Ibuprofen capsules __¥28_ □ Calendar __¥68___ |
| 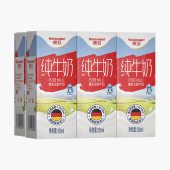 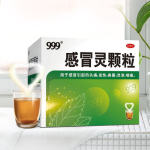 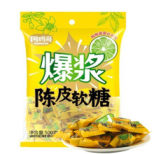 □ Milk __¥19.9__ □ Chinese herb __¥13.3__ □ Candy __¥19.9__ |
| 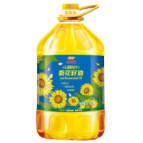 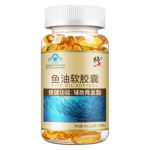 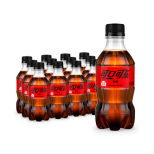 □ Sunflower oil __¥69__ □ Fish oil __¥58__ □ CocaCola __¥25__ |

8. Demographic information

Gender: ○ Male ○ Female

Age:

○ 18 years below

○ 18~25 years old

○ 26~30 years old

○ 31~40 years old

○ 41~50 years old

○ 51~60 years old

○ 60 years above

Education:

○ Middle school and below

○ High school

○ Undergraduate

○ Graduate and above

Monthly income:

○ ¥2000 and below

○ ¥2001-¥4000

○ ¥4001-¥6000

○ ¥6001-¥8000

○ ¥8001-¥10000

○ ¥10001 and above

Monthly expenditure:

○ ¥1000 and below

○ ¥1001-¥3000

○ ¥3001-¥5000

○ ¥5001-¥7000

○ ¥7001-¥9000

○ ¥9001 and above

Please indicate since when you have been quarantining at home:

_________________________________

Please indicate the last time you bought or received allocated groceries:

_________________________________

**Figure S1**. Timeline of COVID-19 Lockdown and Data Collection Periods
