# Supplementary material for: Effects of Perceived Scarcity on Mental Health, Time and Risk Preferences, and Decision-Making During and After COVID-19 Lockdown: Quasi-Natural Experimental Study
Source: JMIR Public Health Surveill. 2025 Aug 29;11:e69496. doi: 10.2196/69496 (PMC12421341; doi:10.2196/69496)
Supplement: Multimedia Appendix 2 [file publichealth-v11-e69496-s002.docx]

**Multimedia Appendix 2**

**Supplementary Tables**

**Table S1.** Test balance of covariates post-matching

| **Variable** | **Mean** | | | **t-test** | |
| --- | --- | --- | --- | --- | --- |
|  | **During** | **After** | **%bias** | **t** | **p>\|t\|** |
| 1.gender | 0.434 | 0.439 | -1.1 | -0.14 | 0.889 |
| 2.age | 0.334 | 0.340 | -1.2 | -0.15 | 0.882 |
| 3.age | 0.127 | 0.128 | -0.6 | -0.07 | 0.947 |
| 4.age | 0.009 | 0.009 | 0.4 | 0.07 | 0.948 |
| 3.edu | 0.048 | 0.051 | -1.3 | -0.17 | 0.865 |
| 4.edu | 0.617 | 0.636 | -4.1 | -0.5 | 0.621 |
| 5.edu | 0.313 | 0.293 | 5 | 0.57 | 0.567 |
| 2.income | 0.039 | 0.043 | -1.9 | -0.22 | 0.826 |
| 3.income | 0.078 | 0.074 | 1.9 | 0.22 | 0.824 |
| 4.income | 0.117 | 0.129 | -3.8 | -0.46 | 0.646 |
| 5.income | 0.193 | 0.196 | -0.8 | -0.11 | 0.914 |
| 6.income | 0.545 | 0.531 | 2.8 | 0.36 | 0.719 |
| 2.expend | 0.199 | 0.186 | 3.3 | 0.43 | 0.67 |
| 3.expend | 0.322 | 0.348 | -5.4 | -0.69 | 0.491 |
| 4.expend | 0.190 | 0.192 | -0.6 | -0.08 | 0.934 |
| 5.expend | 0.102 | 0.094 | 2.6 | 0.37 | 0.709 |
| 6.expend | 0.166 | 0.160 | 1.8 | 0.2 | 0.838 |
| Matching Quality | | | | | |
| Statistic | Value | Threshold |  |  |  |
| Mean % Bias | 2.3 | < 10% |  |  |  |
| Rubin’s B | 8.7% | < 25% |  |  |  |
| Rubin’s R | 0.94 | 0.5–2.0 |  |  |  |

**Figure S1.** Distribution of Propensity Scores Between the “During” and “After” Groups After Matching

**Table S2**. Variance Inflation Factor (VIF) Results Across Models (OLS regressions)

| **Variable** | **VIF (All)** | **VIF (During)** | **VIF (After)** |
| --- | --- | --- | --- |
| Perceived scarcity |  | 15.64 | 2.87 |
| Days in quarantine |  | 16.56 | 8.52 |
| Perceived scarcity # Days in(after) quarantine |  | 2.69 | 6.56 |
| Days since last shopping |  | 4.19 | 5.36 |
| Perceived scarcity # Days since last shopping |  | 3.3 | 5.29 |
| gender | 1.03 | 1.08 | 1.06 |
| age_31-40 | 1.15 | 1.22 | 1.21 |
| age_41-50 | 1.14 | 1.19 | 1.29 |
| age_51+ | 1.15 | 1.23 | 1.15 |
| edu_HighSchool | 3.32 | 3.36 | 3.58 |
| edu_Bachelor | 13.13 | 13.53 | 13.87 |
| edu_Postgrad | 12.69 | 12.93 | 13.5 |
| income_2001-4000 | 2.43 | 2.55 | 2.56 |
| income_4001-6000 | 3.59 | 3.87 | 3.66 |
| income_6001-8000 | 4.98 | 5.21 | 5.2 |
| income_8001-10000 | 6.88 | 7.48 | 6.93 |
| income_10001+ | 10.04 | 11.24 | 10.04 |
| Mean VIF | 4.81 | 6.31 | 5.45 |

**Table S3**. Group differences in main outcomes (During lockdown vs. After lockdown)

|  | During | | After | |  |  |
| --- | --- | --- | --- | --- | --- | --- |
|  | n | mean (SD) | n | mean (SD) | t/z | *P* |
| **Scarcity** |  |  |  |  |  |  |
| Perceived scarcity | 332 | 7.97(2.10) | 339 | 4.35(2.27) | -21.44 | <.001 |
| Days since quarantine | 326 | 31.47(10.10) | 259 | 93.27(24.78) | 40.95 | <.001 |
| Days without shopping | 247 | 6.08(8.52) | 124 | 16.56(25.81) | 5.79 | <.001 |
| **Mental health** |  |  |  |  |  |  |
| Stress | 332 | 2.76(0.70) | 339 | 2.55(0.68) | -3.94 | <.001 |
| Fear of COVID-19 | 332 | 4.05(2.06) | 339 | 4.39(2.11) | 2.09 | .04 |
| **Cognitive functioning** |  |  |  |  |  |  |
| Raven’ Matrices | 332 | 87.00(18.85) | 339 | 87.86(16.34) | 0.63 | .53 |
| **Preference** |  |  |  |  |  |  |
| Proportion of present bias (monetary) | 219 | 0.54(0.50) | 226 | 0.49(0.50) | -1.10 | .27 |
| Proportion of present bias (health) | 219 | 0.47(0.50) | 226 | 0.42(0.50) | -0.97 | .33 |
| Risk aversion (monetary) | 332 | 3.30(1.70) | 339 | 3.11(1.62) | -1.43 | .15 |
| Risk aversion (health) | 332 | 2.19(1.45) | 339 | 2.54(1.47) | 3.58 | <.001 |
| **Purchase decision** |  |  |  |  |  |  |
| Proportion of grocery | 332 | 0.68(0.18) | 339 | 0.64(0.19) | -2.94 | <.001 |
| Proportion of health | 332 | 0.18(0.13) | 339 | 0.19(0.13) | 1.52 | .13 |
| Proportion of temptation | 332 | 0.14(0.15) | 339 | 0.17(0.17) | 2.21 | .03 |

Notes:

^a^*P* values were calculated using two-sided t-tests for continuous variables, a two-sample proportion test for Present Bias with a binary indicator of whether individuals exhibited bias, and the Wilcoxon rank-sum (Mann–Whitney) test for ordinal variables assessing Risk Aversion.

^b^To align with the timing of the lockdown, the analysis of “Days since quarantine” included only those who had quarantined (“During” group, n=326) or had not quarantined (“After” group, n=259) within 120 days. Similarly, the analysis of “Days without shopping” was limited to individuals who had not shopped in the last 90 days for both During (n=247) and After (n=124) groups.

^c^The sample for Present bias was limited to participants ((“During” group, n=219; “After” group, n=226) who consistently chose either the earlier or later payment in both monetary and health time-discounting tasks, switching no more than once between options.

**Table S4**. Tobit regression results for the effects of perceived scarcity and lockdown duration on grocery, health and temptation goods

|  | Proportion of  groceries | | | Proportion of  health items | | | Proportion of  temptation goods | | |
| --- | --- | --- | --- | --- | --- | --- | --- | --- | --- |
|  | b | se | p | b | se | p | b | se | p |
| *Panel A: During lockdown* | | | | | | | | | |
| Perceived scarcity | 0.47 | 0.25 | 0.06 | -0.54 | 0.27 | 0.04 | -0.29 | 0.38 | 0.45 |
| Days in quarantine | -3.74 | 1.48 | 0.01 | 3.72 | 1.70 | 0.03 | 2.13 | 2.12 | 0.31 |
| Perceived scarcity # Days in quarantine | 1.59 | 0.59 | 0.01 | -2.16 | 0.83 | 0.01 | -1.00 | 0.90 | 0.27 |
| Days since last shopping | -1.07 | 0.74 | 0.15 | 0.84 | 0.94 | 0.38 | 1.13 | 1.08 | 0.29 |
| Perceived scarcity # Days since last shopping | 0.79 | 0.58 | 0.18 | -1.36 | 0.81 | 0.09 | -0.51 | 0.86 | 0.56 |
| *Panel B: After lockdown* | | | | | | | | | |
| Perceived scarcity | -0.34 | 0.14 | 0.01 | 0.17 | 0.15 | 0.25 | 0.39 | 0.16 | 0.02 |
| Days after quarantine | -1.34 | 1.09 | 0.22 | 0.21 | 1.26 | 0.87 | 3.61 | 1.58 | 0.02 |
| Perceived scarcity # Days after quarantine | -0.83 | 0.64 | 0.19 | 0.52 | 0.83 | 0.53 | 1.74 | 0.95 | 0.07 |
| Days since last shopping | -0.03 | 0.49 | 0.95 | 0.11 | 0.60 | 0.86 | -0.18 | 0.86 | 0.84 |
| Perceived scarcity # Days since last shopping | -0.35 | 0.35 | 0.32 | 0.42 | 0.44 | 0.33 | 0.20 | 0.61 | 0.74 |

Notes:

^a^Each estimate was obtained from Tobit regression.

^b^All regressions applied propensity score-matching weights to adjust for baseline differences between the groups and controlled demographic variables, including gender, age, education, monthly income and monthly expenditure, to adjust for potential confounding. Robust standard errors were employed across all regressions to ensure the accuracy of the estimates.

**Table S5**. Regression results of lockdown treatment effects by group

|  | Perceived scarcity | | | Stress | | | Fear of COVID-19 | | | Cognitive functioning | | |
| --- | --- | --- | --- | --- | --- | --- | --- | --- | --- | --- | --- | --- |
|  | b | se | p | b | se | p | b | se | p | b | se | p |
| During | 1.28 | 0.06 | <.001 | 0.31 | 0.08 | <.001 | -0.08 | 0.08 | .29 | -0.06 | 0.08 | .49 |
| Constant | -0.80 | 0.32 | .01 | 0.02 | 0.46 | .96 | -0.56 | 0.32 | .08 | -0.07 | 0.43 | .87 |
| Controls | Yes | | | Yes | | | Yes | | | Yes | | |
| Observations | 671 | | | 671 | | | 671 | | | 671 | | |

**Table S5**. Regression results of lockdown treatment effects by group (continued)

|  | Present bias  (monetary) | | | Present bias  (health) | | | Risk aversion (monetary) | | | Risk aversion  (health) | | |
| --- | --- | --- | --- | --- | --- | --- | --- | --- | --- | --- | --- | --- |
|  | b | se | p | b | se | p | b | se | p | b | se | p |
| During | 0.25 | 0.20 | .21 | 0.17 | 0.21 | .40 | 0.13 | 0.14 | .36 | -0.51 | 0.15 | <.001 |
| Constant | -1.03 | 1.18 | .38 | -0.53 | 1.13 | .64 |  |  |  |  |  |  |
| Controls | Yes | | | Yes | | | Yes | | | Yes | | |
| Observations | 445 | | | 445 | | | 671 | | | 671 | | |

**Table S5**. Regression results of lockdown treatment effects by group (continued)

|  | Proportion of  groceries | | | Proportion of  health items | | | Proportion of  temptation goods | | |
| --- | --- | --- | --- | --- | --- | --- | --- | --- | --- |
|  | b | se | p | b | se | p | b | se | p |
| During | 0.24 | 0.08 | <.001 | -0.13 | 0.08 | .10 | -0.17 | 0.08 | .03 |
| Constant | 0.06 | 0.45 | .90 | 0.27 | 0.44 | .54 | -0.29 | 0.35 | .41 |
| Controls | Yes | | | Yes | | | Yes | | |
| Observations | 671 | | | 671 | | | 671 | | |

Notes:

^a^Effects are presented as standardized coefficients with 95% confidence intervals. Each estimate was obtained from OLS regression except for present bias (logistic regression) and risk aversion (ordinal regression).

^b^All regressions applied propensity score-matching weights to adjust for baseline differences between the groups and controlled demographic variables, including gender, age, education, monthly income and monthly expenditure, to adjust for potential confounding. All variables were normalized to z-scores to facilitate comparison. Robust standard errors were employed across all regressions to ensure the accuracy of the estimates.

**Table S6.** Subgroup Analysis

|  | (1) | (2) | (3) | (4) | (5) | (6) | (7) | (8) | (9) | (10) |
| --- | --- | --- | --- | --- | --- | --- | --- | --- | --- | --- |
|  | Stress | Fear of COVID-19 | Cognitive functioning | Present bias (monetary) | Present bias (health) | Risk aversion (monetary) | Risk aversion (health) | Proportion groceries | Proportion health | Proportion temptation |
|  | b/se/p | b/se/p | b/se/p | b/se/p | b/se/p | b/se/p | b/se/p | b/se/p | b/se/p | b/se/p |
| Perceived scarcity | 0.52 | 0.36 | -0.13 | -0.04 | 0.18 | 0.02 | -0.13 | -0.14 | 0.12 | 0.06 |
|  | 0.05 | 0.06 | 0.06 | 0.13 | 0.13 | 0.12 | 0.14 | 0.07 | 0.07 | 0.07 |
|  | 0.00 | 0.00 | 0.02 | 0.76 | 0.18 | 0.89 | 0.34 | 0.04 | 0.06 | 0.34 |
| Female | -0.02 | 0.00 | -0.05 | -0.26 | -0.48 | -0.40 | -0.18 | -0.11 | 0.09 | 0.05 |
|  | 0.07 | 0.07 | 0.08 | 0.16 | 0.16 | 0.14 | 0.15 | 0.08 | 0.08 | 0.08 |
|  | 0.77 | 0.95 | 0.49 | 0.11 | 0.00 | 0.01 | 0.23 | 0.17 | 0.24 | 0.49 |
| Female # Perceived scarcity | -0.07 | 0.06 | 0.02 | 0.03 | -0.14 | -0.06 | 0.11 | 0.16 | -0.15 | -0.07 |
|  | 0.07 | 0.07 | 0.07 | 0.16 | 0.16 | 0.14 | 0.15 | 0.08 | 0.08 | 0.08 |
|  | 0.29 | 0.36 | 0.73 | 0.87 | 0.39 | 0.69 | 0.47 | 0.04 | 0.05 | 0.40 |
| Younger | 0.21 | 0.24 | 0.03 | 0.28 | 0.18 | 0.31 | -0.36 | 0.06 | -0.06 | -0.02 |
|  | 0.15 | 0.16 | 0.18 | 0.35 | 0.35 | 0.29 | 0.31 | 0.16 | 0.17 | 0.16 |
|  | 0.15 | 0.12 | 0.88 | 0.42 | 0.60 | 0.29 | 0.24 | 0.69 | 0.70 | 0.88 |
| Younger # Perceived scarcity | 0.02 | 0.01 | 0.00 | -0.12 | 0.02 | 0.07 | -0.11 | 0.07 | -0.14 | 0.04 |
|  | 0.07 | 0.07 | 0.07 | 0.16 | 0.16 | 0.14 | 0.14 | 0.08 | 0.08 | 0.08 |
|  | 0.75 | 0.89 | 0.97 | 0.45 | 0.92 | 0.60 | 0.44 | 0.40 | 0.06 | 0.61 |
| High school or below | -0.23 | -0.35 | 0.34 | 0.47 | 0.57 | 0.14 | -0.50 | 0.02 | 0.53 | -0.46 |
|  | 0.28 | 0.22 | 0.24 | 0.62 | 0.66 | 0.56 | 0.51 | 0.29 | 0.32 | 0.28 |
|  | 0.42 | 0.11 | 0.16 | 0.45 | 0.39 | 0.80 | 0.33 | 0.96 | 0.10 | 0.10 |
| High school or below # Perceived scarcity | -0.05 | -0.11 | 0.05 | -0.14 | 1.06 | 0.33 | 0.80 | -0.31 | 0.49 | -0.04 |
|  | 0.25 | 0.14 | 0.17 | 0.49 | 0.64 | 0.35 | 0.33 | 0.20 | 0.25 | 0.21 |
|  | 0.83 | 0.42 | 0.78 | 0.77 | 0.10 | 0.35 | 0.01 | 0.11 | 0.05 | 0.86 |
| Lower income (<10000 yuan) | 0.05 | -0.10 | 0.26 | -0.15 | 0.16 | 0.35 | -0.20 | 0.08 | 0.09 | -0.17 |
|  | 0.12 | 0.12 | 0.12 | 0.26 | 0.27 | 0.21 | 0.24 | 0.13 | 0.13 | 0.13 |
|  | 0.67 | 0.40 | 0.04 | 0.57 | 0.55 | 0.11 | 0.40 | 0.53 | 0.50 | 0.18 |
| Lower income (<10000 yuan) # Perceived scarcity | -0.07 | -0.10 | 0.13 | -0.18 | -0.26 | 0.25 | 0.09 | 0.02 | -0.09 | 0.05 |
|  | 0.07 | 0.07 | 0.08 | 0.16 | 0.17 | 0.14 | 0.15 | 0.08 | 0.08 | 0.08 |
|  | 0.30 | 0.13 | 0.09 | 0.28 | 0.12 | 0.08 | 0.54 | 0.81 | 0.25 | 0.52 |
| Lower expenditure (<5000 yuan) | 0.14 | 0.06 | 0.03 | -0.14 | -0.02 | 0.45 | 0.19 | 0.16 | -0.18 | -0.04 |
|  | 0.13 | 0.14 | 0.15 | 0.29 | 0.30 | 0.26 | 0.26 | 0.15 | 0.15 | 0.14 |
|  | 0.28 | 0.66 | 0.82 | 0.64 | 0.96 | 0.08 | 0.45 | 0.27 | 0.22 | 0.79 |
| Lower expenditure (<5000 yuan) # Perceived scarcity | -0.07 | 0.00 | -0.01 | -0.37 | 0.05 | -0.05 | -0.22 | -0.02 | 0.02 | 0.00 |
|  | 0.07 | 0.07 | 0.07 | 0.16 | 0.16 | 0.14 | 0.15 | 0.08 | 0.08 | 0.08 |
|  | 0.32 | 0.99 | 0.90 | 0.02 | 0.76 | 0.72 | 0.14 | 0.81 | 0.78 | 0.96 |
| Observations | 671 | 671 | 671 | 671 | 671 | 671 | 671 | 671 | 671 | 671 |

|  | Section/topic | No | CONSORT 2025 checklist item description | Reported on page no. |
| --- | --- | --- | --- | --- |
|  | **Title and abstract** | | |  |
|  | Title and structured abstract | 1a | Identification as a randomised trial | Page 1 |
|  |  | 1b | Structured summary of the trial design, methods, results, and conclusions | Page 1 |
|  | **Open science** | | |  |
|  | Trial registration | 2 | Name of trial registry, identifying number (with URL) and date of registration | Not applicable |
|  | Protocol and statistical analysis plan | 3 | Where the trial protocol and statistical analysis plan can be accessed | Not applicable |
|  | Data sharing | 4 | Where and how the individual de-identified participant data (including data dictionary), statistical code and any other materials can be accessed | Page 19 |
|  | Funding and conflicts of interest | 5a | Sources of funding and other support (eg, supply of drugs), and role of funders in the design, conduct, analysis and reporting of the trial | Page 18 |
|  |  | 5b | Financial and other conflicts of interest of the manuscript authors | Page 19 |
|  | **Introduction** | | |  |
|  | Background and rationale | 6 | Scientific background and rationale | Pages 2–4 |
|  | Objectives | 7 | Specific objectives related to benefits and harms | Pages 3-4 |
|  | **Methods** | | |  |
|  | Patient and public involvement | 8 | Details of patient or public involvement in the design, conduct and reporting of the trial | Not applicable |
|  | Trial design | 9 | Description of trial design including type of trial (eg, parallel group, crossover), allocation ratio, and framework (eg, superiority, equivalence, non-inferiority, exploratory) | Page 4 |
|  | Changes to trial protocol | 10 | Important changes to the trial after it commenced including any outcomes or analyses that were not prespecified, with reason | Not applicable |
|  | Trial setting | 11 | Settings (eg, community, hospital) and locations (eg, countries, sites) where the trial was conducted | Page 4-5 |
|  | Eligibility criteria | 12a | Eligibility criteria for participants | Page 5 |
|  |  | 12b | If applicable, eligibility criteria for sites and for individuals delivering the interventions (eg, surgeons, physiotherapists) | Not applicable |
|  | Intervention and comparator | 13 | Intervention and comparator with sufficient details to allow replication. If relevant, where additional materials describing the intervention and comparator (eg, intervention manual) can be accessed | Page 4-5 |
|  | Outcomes | 14 | Prespecified primary and secondary outcomes, including the specific measurement variable (eg, systolic blood pressure), analysis metric (eg, change from baseline, final value, time to event), method of aggregation (eg, median, proportion), and time point for each outcome | Page 6-7 |
|  | Harms | 15 | How harms were defined and assessed (eg, systematically, non-systematically) | Not applicable |
|  | Sample size | 16a | How sample size was determined, including all assumptions supporting the sample size calculation | Page 5 |
|  |  | 16b | Explanation of any interim analyses and stopping guidelines | Page 5 |
|  | Randomisation: |  |  |  |
|  | Sequence generation | 17a | Who generated the random allocation sequence and the method used | Page 4 |
|  |  | 17b | Type of randomisation and details of any restriction (eg, stratification, blocking and block size) | Page 4 |
|  |  |  |  | **Reported on page no.** |
|  | Allocation concealment mechanism | 18 | Mechanism used to implement the random allocation sequence (eg, central computer/telephone; sequentially numbered, opaque, sealed containers), describing any steps to conceal the sequence until interventions were assigned | Not applicable |
|  | Implementation | 19 | Whether the personnel who enrolled and those who assigned participants to the interventions had access to the random allocation sequence | Not applicable |
|  | Blinding | 20a | Who was blinded after assignment to interventions (eg, participants, care providers, outcome assessors, data analysts) | Not applicable |
|  |  | 20b | If blinded, how blinding was achieved and description of the similarity of interventions | Not applicable |
|  | Statistical methods | 21a | Statistical methods used to compare groups for primary and secondary outcomes, including harms | Page 7 |
|  |  | 21b | Definition of who is included in each analysis (eg, all randomised participants), and in which group | Page 5 |
|  |  | 21c | How missing data were handled in the analysis | Page 5 |
|  |  | 21d | Methods for any additional analyses (eg, subgroup and sensitivity analyses), distinguishing prespecified from post hoc | Page 7 |
|  | **Results** | | |  |
|  | Participant flow, including flow diagram | 22a | For each group, the numbers of participants who were randomly assigned, received intended intervention, and were analysed for the primary outcome | Page 5-6 |
|  |  | 22b | For each group, losses and exclusions after randomisation, together with reasons | Page 5 |
|  | Recruitment | 23a | Dates defining the periods of recruitment and follow-up for outcomes of benefits and harms | Page 4 |
|  |  | 23b | If relevant, why the trial ended or was stopped | Page 4 |
|  | Intervention and comparator delivery | 24a | Intervention and comparator as they were actually administered (eg, where appropriate, who delivered the intervention/comparator, how participants adhered, whether they were delivered as intended (fidelity)) | Not applicable |
|  |  | 24b | Concomitant care received during the trial for each group | Not applicable |
|  | Baseline data | 25 | A table showing baseline demographic and clinical characteristics for each group | Page 9 |
|  | Numbers analysed,  outcomes and estimation | 26 | For each primary and secondary outcome, by group:  ● the number of participants included in the analysis  ● the number of participants with available data at the outcome time point  ● result for each group, and the estimated effect size and its precision (such as 95% confidence interval)  ● for binary outcomes, presentation of both absolute and relative effect size | Page 10-14 |
|  | Harms | 27 | All harms or unintended events in each group | Not applicable |
|  | Ancillary analyses | 28 | Any other analyses performed, including subgroup and sensitivity analyses, distinguishing pre-specified from post hoc | Appendix 2, Page 2 |
|  | **Discussion** | | |  |
|  | Interpretation | 29 | Interpretation consistent with results, balancing benefits and harms, and considering other relevant evidence | Page 14-17 |
|  | Limitations | 30 | Trial limitations, addressing sources of potential bias, imprecision, generalisability, and, if relevant, multiplicity of analyses | Page 17-18 |

Citation: Hopewell S, Chan AW, Collins GS, Hróbjartsson A, Moher D, Schulz KF, et al. CONSORT 2025 Statement: updated guideline for reporting randomised trials. BMJ. 2025; 388:e081123. <https://dx.doi.org/10.1136/bmj-2024-081123>
© 2025 Hopewell et al. This is an Open Access article distributed under the terms of the Creative Commons Attribution License (<https://creativecommons.org/licenses/by/4.0/>), which permits unrestricted use, distribution, and reproduction in any medium, provided the original work is properly cited.

*We strongly recommend reading this statement in conjunction with the CONSORT 2025 Explanation and Elaboration and/or the CONSORT 2025 Expanded Checklist for important clarifications on all the items. We also recommend reading relevant CONSORT extensions. See [www.consort-spirit.org](http://www.consort-spirit.org).
